# Pathway-instructed therapeutic selection of ruxolitinib reduces neuroinflammation in fungal postinfectious inflammatory syndrome

**DOI:** 10.1126/sciadv.adi9885

**Published:** 2025-03-21

**Authors:** Jessica C. Hargarten, Kenneth Ssebambulidde, Seher H. Anjum, Malcolm J. Vaughan, Jintao Xu, Anutosh Ganguly, Brittany Dulek, Francisco Otaizo-Carrasquero, Brian Song, Sijia Tao, Yoon-Dong Park, Terri L. Scott, Tracey-Ann Höltermann, Raymond F. Schinazi, Prashant Chittiboina, Bridgette Jeanne Billioux, Dima A. Hammoud, Michal A. Olszewski, Peter R. Williamson

**Affiliations:** ^1^ Laboratory of Clinical Immunology and Microbiology (LCIM), Division of Intramural Research (DIR), National Institute of Allergy and Infectious Diseases (NIAID), National Institutes of Health (NIH), Bethesda, MD, USA.; ^2^ Infectious Diseases Institute, College of Health Sciences, Makerere University, Kampala, Uganda.; ^3^ Division of Pulmonary and Critical Care Medicine, Department of Internal Medicine, University of Michigan Health System, Ann Arbor, MI, USA.; ^4^ Research Service, Ann Arbor Veterans Affairs (VA) Healthcare System, Department of Veterans Affairs Health System, Ann Arbor, MI, USA.; ^5^ Integrated Data Science Section, NIAID, NIH, Bethesda, MD, USA.; ^6^ Genomic Research Section, Research Technologies Branch, NIAID, NIH, Bethesda, MD, USA.; ^7^ Center for Viroscience and Cure, Laboratory of Biochemical Pharmacology, Department of Pediatrics, Emory University School ofMedicine and Children‘s Healthcare of Atlanta, Atlanta, GA, USA.; ^8^ Center for Acquired Immunodeficiency Syndrome (AIDS) Research, Department of Pediatrics, School of Medicine, Emory University, Atlanta, GA, USA.; ^9^ Surgical Neurology Branch, National Institute of Neurological Disorders and Stroke (NINDS), NIH, Bethesda, MD, USA.; ^10^ Section of Infections of the Nervous System, NINDS, NIH, Bethesda, MD, USA.; ^11^ Center for Infectious Disease Imaging (CIDI), Radiology and Imaging Sciences, Clinical Center, NIH, Bethesda, MD, USA.

## Abstract

Therapies to reduce neuroinflammation following resolution of acute central nervous system (CNS) infections are urgently needed, particularly for patients with non–HIV-associated cryptococcal meningoencephalitis complicated by a postinfectious inflammatory response syndrome (cPIIRS). To identify druggable targets in cPIIRS, patient cerebral spinal fluid samples underwent transcriptional analysis, revealing a Janus kinase/signal transducer and activator of transcription (JAK/STAT) pathway dominance in neuroinflammatory gene signatures. MurinecPIIRS models recapitulated this pathway predominance and treatment with the JAK inhibitor ruxolitinib, confirmed a mechanistic requirement for this pathway in disease pathology. Ruxolitinib treatment improved markers of neuronal damage, reduced activated T cell and myeloid cells, and improved weight. On the basis of these findings, we conducted a first-in-human ruxolitinib treatment of patients with cPIIRS (NCT00001352). Ruxolitinib treatment of six patients led to demonstrated tolerability, reductions in inflammatory biomarkers and activated immune cells, and improved brain imaging. These results advocate for pathway-instructed therapeutics in neuroinflammatory diseases and endorse JAK inhibitors in further clinical studies of cPIIRS.

## Teaser

Pathway-instructed therapy identified ruxolitinib as a promising treatment for a postinfectious neuroinflammatory syndrome.

## INTRODUCTION

Postinfection inflammatory sequalae have recently gained considerable attention following bacterial and viral meningitides, including postacute sequelae of severe acute respiratory syndrome coronavirus 2 infection (*1*, *2*). Recently, neurological inflammatory syndromes have been described in fungal infections principally with the neurotropic fungus *Cryptococcus* (*Cn*) and include HIV-related immune reconstitution inflammatory syndromes (cIRIS) (*3*) and, more recently, non–HIV-related inflammatory response syndromes (cPIIRS) (*4*, *5*). Cryptococcal meningoencephalitis (CM) currently accounts for the largest number of nonviral meningitides in the US (*6*), and neurological inflammatory syndromes are important causes of morbidity and mortality in all susceptible hosts. The skull’s rigid structure limits unchecked tissue expansion from inflammatory-induced edema. Subsequent compression results in neurological deterioration or even fatal uncal herniation because of rising intracranial pressure (*7*). Distinct from cIRIS, clinical deterioration in cPIIRS manifests without clear immune reconstitution and likely arises from antigen despite negative cerebral spinal fluid (CSF) cultures (*4*). Cohort studies have found pulse-corticosteroid taper therapy effective against this inflammation, leading to prompt clinical improvement in all treated patients thus far (*8*). Symptom alleviation has been linked to reductions in multiple CSF inflammatory markers, including activated HLA-DR^+^ CD4^+^ and CD8^+^ T cells, inflammatory monocytes, soluble CD25 (IL-2R), interleukin-6 (IL-6), and protein levels with associated increases in glucose (*8*), a marker of intrathecal oxidative metabolism. Most patients diagnosed with cPIIRS require corticosteroid therapy for a period of a year (*6*). This steroid treatment taper rate is notably slower compared to conditions like cIRIS and tuberculous meningitis (*9*), leading to potentially increased rates of steroid-related complications (*10*). However, progress in developing effective, rationally targeted therapies for cPIIRS has been limited by our lack of detailed mechanistic understanding of the underlying key inflammatory pathways in cPIIRS.

Thus, to uncover molecular mechanisms involved in the pathogenesis of cPIIRS and to inform the prioritization of new therapeutic options, the present study used bioinformatics to identify the major upstream transcriptional regulators driving the pathogenesis of cPIIRS and, on the basis of these findings, tested a specific inhibitor of the predominant pathway first in a murine cPIIRS model and second in a small cohort of patients with cPIIRS. Our studies presented here demonstrated the following: (i) signal transducer and activator of transcription 1 (STAT1)– and STAT3-associated downstream genes are prominent in cPIIRS; (ii) the Janus kinase (JAK)/STAT inhibitor ruxolitinib alone led to significant reductions in JAK/STAT transcriptional targets in a murine cPIIRS model and demonstrated weight gains, reductions in central nervous system (CNS) lesion size, and improvements in markers of brain function; and (iii) adjunctive ruxolitinib combined with the standard-of-care antifungal drug amphotericin B (AMB) in six patients was safe and yielded therapeutic reductions in CNS-associated symptoms and patient function and (iv) resulted in significant reductions in patient CNS inflammation and magnetic resonance imaging (MRI) abnormalities in seven treatment courses of six patients. In summary, these findings support a prominent role for the JAK/STAT pathway in the pathogenesis of cPIIRS, support the concept of pathway-instructed therapy to accelerate treatment discovery, and provide a proof of concept for the further study of ruxolitinib as a potential adjuvant therapy of cPIIRS and possibly other types of CNS inflammatory conditions triggered by infections.

## RESULTS

### JAK/STAT pathway prominence in cPIIRS prioritizes the therapeutic candidate to reduce inflammation

**Fig. 1.**
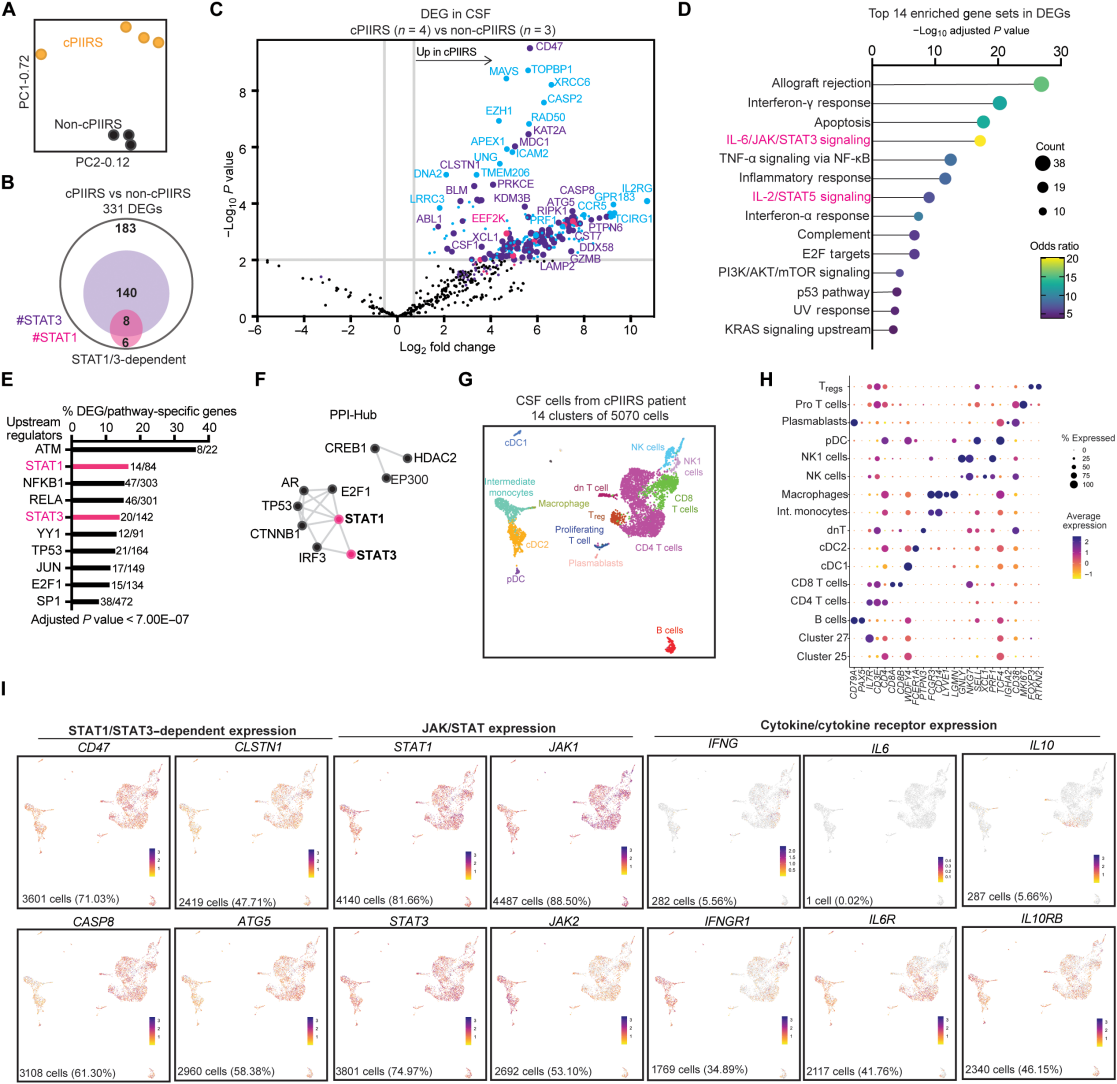
Transcriptional pathway analysis identifies the JAK/STAT inflammatory pathway as predominant in the CSF of patients with cPIIRS. (**A** to **F**) Transcriptional analysis of the CFS of patients with cPIIRS (at diagnosis) and non-cPIIRS donor CSF was performed using a NanoString multiplex neuroinflammation panel (*n* = 4 patients and 3 donors from one experiment). (A) PCA of normalized read counts of 757 total transcripts associated with neuroinflammation for the CSF of patients with cPIIRS and non-cPIIRS donor CSF. (B) Venn diagram indicating the number of DEGs (as described in Methods) in CSF known to be STAT1 (pink) or STAT3 (purple) dependent. Underlined are the total DEGs for each comparison. (C) Scatterplot of NanoString analysis comparing gene expression profiles of CSF of patients with cPIIRS to non-cPIIRS donor CSF. Highlighted are STAT1 (pink)/STAT3 (purple)–dependent and independent (light blue) DEGs. (D) Top 14 significantly enriched biological pathways identified in the CSF of patients with cPIIRS (MSigDB Hallmark 2020). (E and F) Pathway analysis using DEGs between the CSF of patients with cPIIRS and non-cPIIRS donor CSF was performed using Enrichr TRRUST Transcription Factors 2019 Database (E) and the Enrichr Transcription Factor PPI to construct networks (F). (**G** to **I**) CSF cells from a patient (at cPIIRS diagnosis, before treatment) were subjected to scRNA-seq. (G) UMAP plot of CSF cell scRNA-seq data showing cell-type clusters used for subsequent analysis (*n* = 5070). (H) Dotplot depicting selected marker genes in cell clusters. The dot size corresponds to the percentage of cells expressing the gene, and the color indicates the average per cell gene expression. (I) Feature plots of STAT1/STAT3–dependent genes, JAK/STAT genes, and cytokine/cognate cytokine receptor genes using colors to indicate gene expression (log_2_UMI counts) levels in the UMAP embedding from (G). The number and percent of cells with detected expression for each gene are reported at the bottom of each feature plot.

To identify the optimal therapeutic targets responsible for inflammatory processes in cPIIRS, we evaluated changes in RNA transcripts in cells isolated from the CSF of four patients at the time of cPIIRS diagnosis and three non-cPIIRS donors using a human 757 gene NanoString neuroinflammation multiplex panel (table S1) (*11*–*13*). Principal component analysis (PCA) demonstrated that the CSF of patients with cPIIRS and non-cPIIRS donor CSF formed separate clusters (Fig. 1A), and 148 genes were found to be differentially expressed (Fig. 1, B and C). The CSF of patients with cPIIRS showed elevated pathway scores for neuroinflammation pathways analyzed compared to non-cPIIRS donor CSF, particularly for adaptive immune responses, cytokine signaling, and inflammation (fig. S1A). Using gene-set enrichment analysis, we found “IL-6/JAK/STAT3 Signaling” among the top four enriched hallmark pathways curated from the Molecular Signatures Database (Fig. 1D) (*14*), with 44% of these differentially expressed genes (DEGs) being JAK/STAT signaling dependent (Fig. 1B and table S2). Upstream transcription factor analyses identified JAK/STAT signaling, particularly STAT1 and STAT3, in the top five regulators with high significance (Fig. 1E) and predicted that AR, TP53, IRG3, E2F1, and CTNNB1 interact with STAT1 and STAT3 to illicit the changes in DEGs (Fig. 1F). Consistent with these results, “IFN-γ response,” which is downstream and dependent on JAK/STAT signaling, was identified as the top enriched hallmark pathway (Fig. 1D).

To define the cellular populations associated with this JAK/STAT signature, we generated single-cell RNA sequencing (scRNA-seq) data from the CSF of a previously healthy patient at the time of cPIIRS diagnosis and before treatment and obtained 5070 CSF single-cell transcriptomes classified into 14 final cell clusters (Fig. 1G). On the basis of marker gene expression (Fig. 1H and fig. S1C), we identified αβ T cells (*CD3E*) subsetting into CD4+ T cells (*CD4* and *IL7R*), CD8+ T cells (*CD8A*, *CD8B*, and *NKG7*), and regulatory T cells (*FOXP3* and *RTKN2*); two natural killer (NK) cell clusters (*NKG7* and *PRF1*) most likely represented by the more cytotoxic and mature CD56dim (NK1; *FCGR3A/*CD16 and *GNLY*) and unique NK (NK cell; *SELL*/CD62L, *XCL1*, and *CD38*) subsets; B cell clusters (*CD79A*, *PAX5*, and *IGH* gene family) and plasmablasts (*CD79A*, *CD38*, and *IGHA2*; negative for *MS4A1*/CD20); and myeloid lineage cells (*LYZ*) separated into cDC type 1 (cDC1; *WDFY4*, *XCR1*, and *BATF3*), cDC type 2 (cDC2; *FCER1A*, *CD1C*, *ST18*, and *CLEC10A*), intermediate monocytes (Int. monocytes; *CD14* and *SELL*), and macrophages (*LYVE*, *LGMN*, and *CD14*). Additional clusters represented plasmacytoid dendritic cells (*TCF4*/E2-2 and *TNFRSF21*/DR6), proliferating T cells (*MKI67*), and double-negative T cells (*PTPN3*).

We next analyzed these cell clusters to identify specific cells associated with the JAK/STAT–dependent gene signature. We found that CSF cells in every identified cell cluster were positive for the following: (i) STAT1/STAT3–dependent gene expression (*CD47*, *CLSTN1*, *CASP8*, and *ATG5*); (ii) expression of *JAK1*, *JAK2*, *STAT1*, and *STAT3* genes themselves; and (iii) expression of an array of cytokine receptors that requires JAK/STAT signaling downstream of cytokine ligation (Fig. 1I) (*15*) and likely targets for JAK/STAT inhibition. Pro-inflammatory cytokine (*IL6* and *IFNG*) expression by CSF cells was minimal, whereas expression of the counter-regulatory cytokine *IL10* was notable in the CD4+ T cell cluster. Thus, the prominence of JAK/STAT signaling in the NanoString transcriptional signatures and the breadth of cell populations involved strongly support the predominant role of JAK/STAT in cPIIRS pathology in humans.

### Murine cPIIRS is associated with a prominence of JAK/STAT regulatory targets

**Fig. 2.**
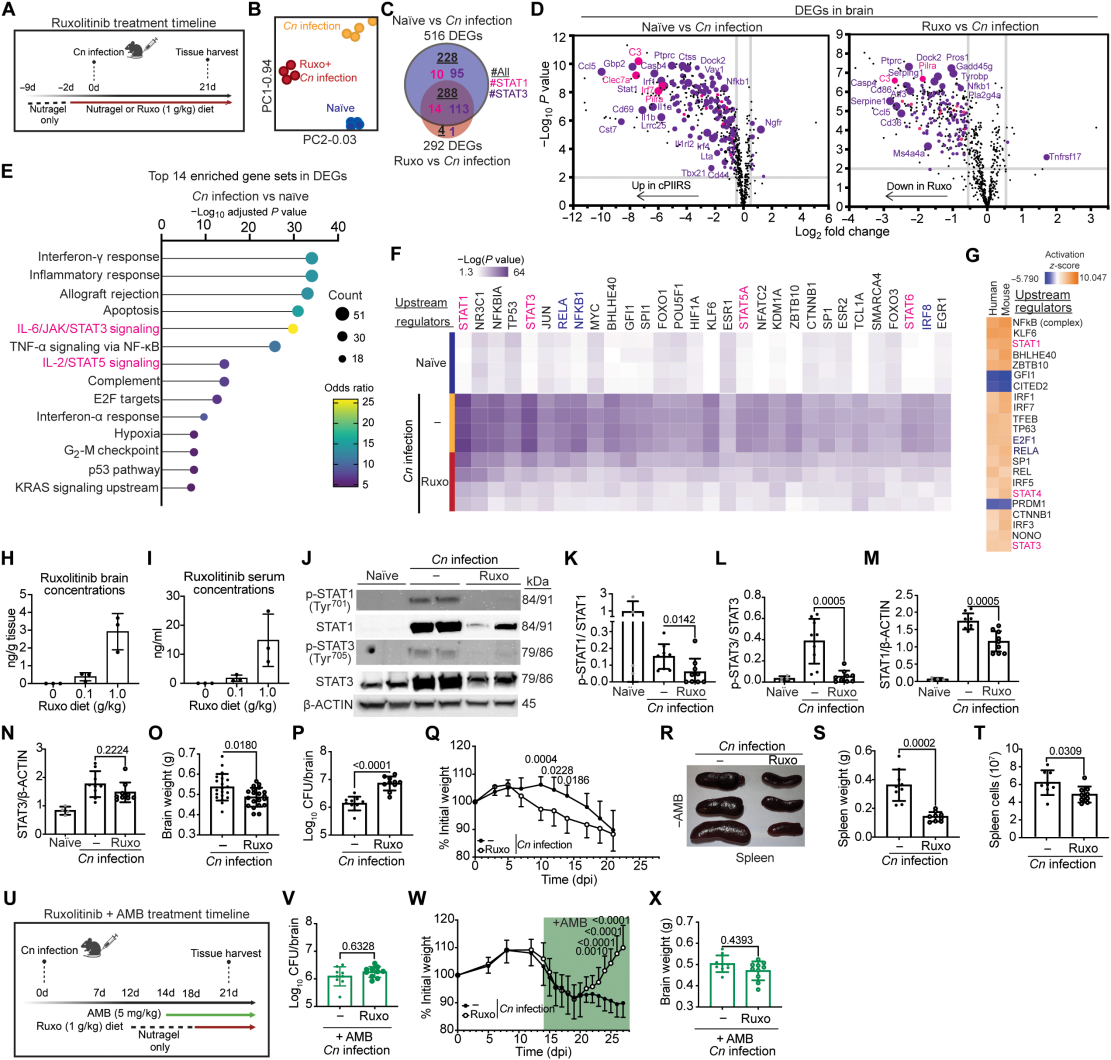
Transcriptional pathway analysis identifies the JAK/STAT inflammatory pathway as predominant in a cPIIRS mouse model and disease improvement with ruxolitinib. (**A**) Graphical schema for the mouse cPIIRS model and daily treatment with ruxolitinib. d, days. (**B** to **G**) Gene transcription of mouse brains at 21 dpi was analyzed using a NanoString Neuroinflammation panel (*n* = 4 mice per group). (B) PCA of mouse brain NanoString results. (C) Venn diagram showing overlapping DEGs in the mouse brain, including STAT1 (pink)–dependent, STAT3 (purple)–dependent, and total (underlined) DEGs. (D) Scatterplots comparing gene expression profiles in mouse brains. Highlighted are STAT1 (pink)– and STAT3 (purple)–dependent DEGs. (E) Top 14 significantly enriched biological pathways identified in the mouse brain (MSigDB Hallmark 2020). (F) Top upstream regulators of DEGs in mouse brains (IPA). Highlighted are JAK/STAT pathway–specific (pink) and consensus downstream JAK/STAT targets (blue). (G) Top IPA-predicted upstream regulators of DEGs in humans (patients with cPIIRS versus non-cPIIRS donors) and mice (*Cn* infection versus naïve). (**H** and **I**) Ruxolitinib concentrations in the mouse brain at 21 dpi (H) and serum (I) (*n* = 3 mice per group). (**J** to **N**) Immunoblot analysis of phospho-STAT1 (Tyr^701^) [(J) and (K)], phospho-STAT3 (Tyr^705^) [(J) and (L)], total STAT1 [(J) and (M)], and total STAT3 [(J) and (N)] levels in mouse brains (*n* = 4 to 8 mice per group; two experiments). (**O** to **T**) Brain weight (21 dpi) (O), brain fungal burden (21 dpi) (P), mouse weights (throughout infection) (Q), spleen size (21 dpi) (R), spleen weight (21 dpi) (S), and splenocytes (21 dpi) (T) were enumerated. CFU, colony-forming units. (**U**) Graphical schema for the mouse cPIIRS model with AMB/ruxolitinib treatment. (**V** to **X**) Brain fungal burden (21 dpi) (V), mouse weights (W), and brain weight (21 dpi) (X) were enumerated. Error bars indicate SD from two independent experiments (*n* = 8 to 20 mice per time point). Student’s *t* test, with *P* values indicated above each comparison. Graphical schemas were created with BioRender.com.

To further delineate this hypothesis, we used a recently described cPIIRS mouse model (Fig. 2A) (*16*) that would allow for the study of cPIIRS in the absence of confounding therapeutic agents and provide preclinical insight to therapeutic selection. In this model, *Cn* rapidly enters and expands within the murine CNS within a few days postinfection (dpi) but does not initially induce an inflammatory response at this site unlike other models (*17*–*22*). Not until ~21 dpi and during the onset of fungal clearance does recruitment of pathogenic T helper 1–polarized CD4^+^ T cells lead to CNS pathology and a cPIIRS-like phenotype (elevated CD4+ T cells and other inflammatory cell populations, CNS cytokinemia, and decreased mental status) as depletion of CD4^+^ T cells in this model prevents signs of disease and mortality. Using this model, we first asked whether a predominant JAK/STAT pathway signature is detected in the brain of mice at peak inflammation (21 dpi) using a mouse NanoString Neuroinflammation panel, which targets 757 genes involved in neuroimmune processes and interactions (*11*–*13*, *23*, *24*). Transcriptionally, the brains of naïve mice and *Cn*-infected mice clustered independently (Fig. 2B) and 516 genes were found to be differentially expressed (Fig. 2, C and D). *Cn*-infected mice showed elevated pathway scores for all neuroinflammation pathways analyzed compared to naïve mice (fig. S2A). However, as seen with human cPIIRS CSF samples, cPIIRS mouse brains showed “IL-6/JAK/STAT3 Signaling” pathway enrichment (Fig. 2E) (*14*), with 45% of these DEGs being JAK/STAT signaling dependent (Fig. 2, C and D, and table S3). Ingenuity pathway analysis (IPA) identified STAT1 and STAT3 in the top five transcriptional regulators with high significance (Fig. 2F and fig. S2, B and C). Notably, strong similarities were observed between the mouse (*Cn* infection versus naïve) and human (cPIIRS versus non-cPIIRS) cPIIRS DEG profiles with STAT1 and NF-κB (nuclear factor κB) in the top three IPA-predicted upstream regulators of DEGs (Fig. 2G), underscoring the applicability of the murine PIIRS model to human pathology.

To determine whether pharmacological inhibition of JAK1 and JAK2 with a Food and Drug Administration (FDA)–approved pharmaceutical would attenuate cPIIRS disease severity, *Cn*-infected mice were treated with either ruxolitinib (1 g ruxolitinib/kg Nutragel daily) or vehicle (Nutragel) according to previously described ruxolitinib therapeutic models (*25*, *26*). At 21 dpi, ruxolitinib was readily detected in serum and brains of all treated animals, achieving median steady-state brain and serum levels of 3.3 ng/g (*n* = 3 mice; Fig. 2H) and 12.1 ng/ml (*n* = 3 mice; Fig. 2I). [Mice who received 1/10th the efficacious dose of ruxolitinib achieved a median level of 0.5 ng/g brain (*n* = 3 mice; Fig. 2H) and 1.4 ng/ml serum (*n* = 3 mice; Fig. 2I).] The higher dose was effective as the JAK/STAT transcriptional signature (Fig. 2, D and F), and STAT1 and STAT3 phosphorylation (Fig. 2, J to N) was reduced within the brains of treated mice. Notably, JAK/STAT inhibition with ruxolitinib treatment led to a reduction in cerebral edema, as measured by brain weight (Fig. 2O), but increased fungal burdens (Fig. 2P), resulting in more pronounced weight loss between 10 and 14 dpi (Fig. 2Q). Consistent with a systemic effect on inflammation, spleen size, weights (Fig. 2, R and S, and fig. S2D), and splenocyte numbers (Fig. 2T) showed reductions after treatment. The addition of the antifungal agent AMB (Fig. 2U), per standards of patient care, reduced and normalized brain fungal loads between the treatment groups (Fig. 2V), leading to marked and significant weight gains in ruxolitinib-treated mice (Fig. 2W) but without further reductions in brain weights, likely due to reduced antigen load resulting from antifungal therapy (Fig. 2X). Together, these data demonstrate the prominence of JAK/STAT pathway activation in mediating cPIIRS in mice in the absence of corticosteroids and the efficacy of ruxolitinib treatment in mouse models of cPIIRS.

### JAK/STAT inhibition protects cPIIRS mice from neuronal cell apoptotic death

**Fig. 3.**
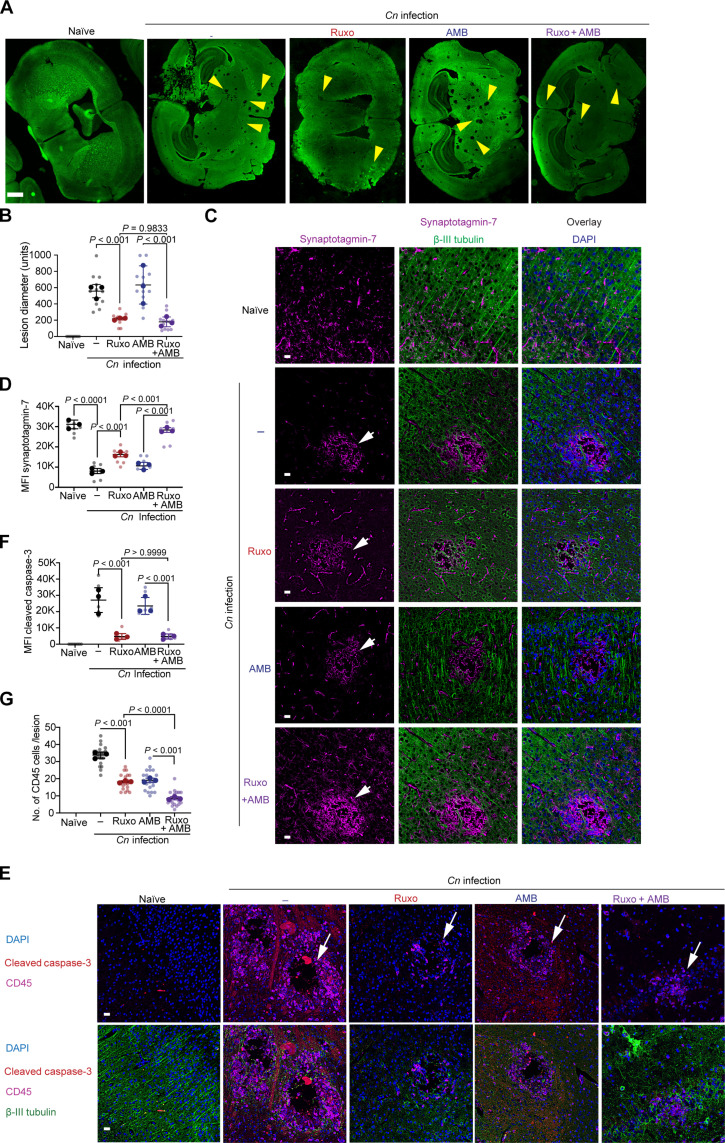
JAK/STAT–mediated inflammatory neurotoxicity is mitigated by ruxolitinib in a mouse model of cPIIRS. (**A**) IFM of brain sections stained with antibodies to β-III tubulin (green) was used to assess cryptococcal lesion size as indicated (yellow arrows). Scale bar, 100 μm. (**B**) Summary statistics of brain lesion sizes from Fig. 2A. (**C**) IFM of the brain sections stained with antibodies to β-III tubulin (green), Syt-7 (red), and 4′,6-diamidino-2-phenylindole (DAPI; blue). Cryptococcal lesion indicated (white arrow). Scale bars, 10 μm. (**D**) Summary statistics of the MFI quantitation of Syt-7 from Fig. 2C. (**E**) IFM of brain sections stained with β-III tubulin (green), cleaved caspase-3 (red), CD45 (pink), and DAPI (blue). Cryptococcal lesion indicated (white arrow). Scale bars, 10 μm. (**F** and **G**) Summary statistics of cleaved caspase-3 MFI (F) and CD45^+^ cells/lesion (G) from Fig. 2E. Data shown are representative of two independent experiments (*n* = 3 mice per group) +/− SEM, and at least 10 fields were examined for each sample. Statistical significance was determined using the linear regression models fit via GEE with *P* values indicated over each comparison.

To elucidate the mechanisms underlying these findings and characterize the neuropathology specifically resulting from excessive JAK/STAT activation in cPIIRS, we measured the size of cryptococcal lesions within brains during onset of inflammation and after inhibition with ruxolitinib. The use of a specific JAK1/2 inhibitor in mice allowed time and dose modulation to reduce effects on fungal loads and facilitate preclinical development. All infected mice developed “Swiss cheese”–like lesions representing *Cn* pseudocyst formation (Fig. 3A, yellow arrows). Ruxolitinib-treated mice displayed lesions that were ~50% smaller in diameter compared to the other infected groups, either not treated with antifungals or treated with AMB (Fig. 3B). The persistence of the protective effect of ruxolitinib after reduction and normalization of fungal load with AMB supports the finding that the residual inflammation is damaging even after fungal load reductions from antifungal therapy, similar to human cPIIRS after antifungal therapy (*4*). To determine how treatments modulate the development of neuronal injury and whether it could prevent *Cn*/inflammation–induced neurological damage in cPIIRS, we assessed expression of a protein integral to neurotransmission, synaptotagmin-7 (Syt7), at the perimeter of cryptococcal lesions by immunofluorescence microscopy (IFM) (*27*). Our IFM data revealed a stark reduction of Syt7 expression in neuronal tissue (visualized by β-III tubulin staining) at the perimeter of the cryptococcal pseudocysts compared to the background expression of Syt7 in similar locations in brains of naïve mice (Fig. 3, C and D, and fig. S3). Treatment with ruxolitinib, but not AMB alone, led to a partial recovery of Syt7 expression. However, the combined treatment of ruxolitinib and AMB restored Syt7 expression levels to the baseline, suggesting a synergistic, protective effect when both drugs were used. This model previously demonstrated neuronal apoptosis at the perimeter of the cryptococcal microcysts linked to pathological inflammation (*27*, *28*). Treatment was also effective at preventing neuronal apoptosis (an important pathway in cPIIRS suggested by Figs. 1D and 2E), assessed by visualizing cleaved caspase-3 within β-III tubulin–positive neurons (Fig. 3E). The β-III tubulin–positive neuronal bundles were negative for the cleaved caspase-3 signal in naïve mouse brains; however, in the cPIIRS mice, there was a significant presence of cleaved caspase-3 signals, clearly localized with neurons but not CD45^+^ immune cells. Treatment with ruxolitinib alone or in conjunction with AMB led to a significant reduction of cleaved caspase-3 signals within neurons (Fig. 3F). Thus, the inflammatory processes rather than fungal burden appeared to be the major driver of lesion enlargement. Lesions contained significant numbers of CD45 immune cells per lesion, which were reduced after ruxolitinib treatment (Fig. 3G). In conclusion, our findings support the notion that inflammation supported by JAK/STAT signaling is an important driver of cPIIRS CNS pathology and its inhibition with ruxolitinib offers protection from neuronal apoptotic cell death and preserves neuronal connectivity.

### PCA of cPIIRS murine brain inflammation demonstrates JAK/STAT–driven immune activation

**Fig. 4.**
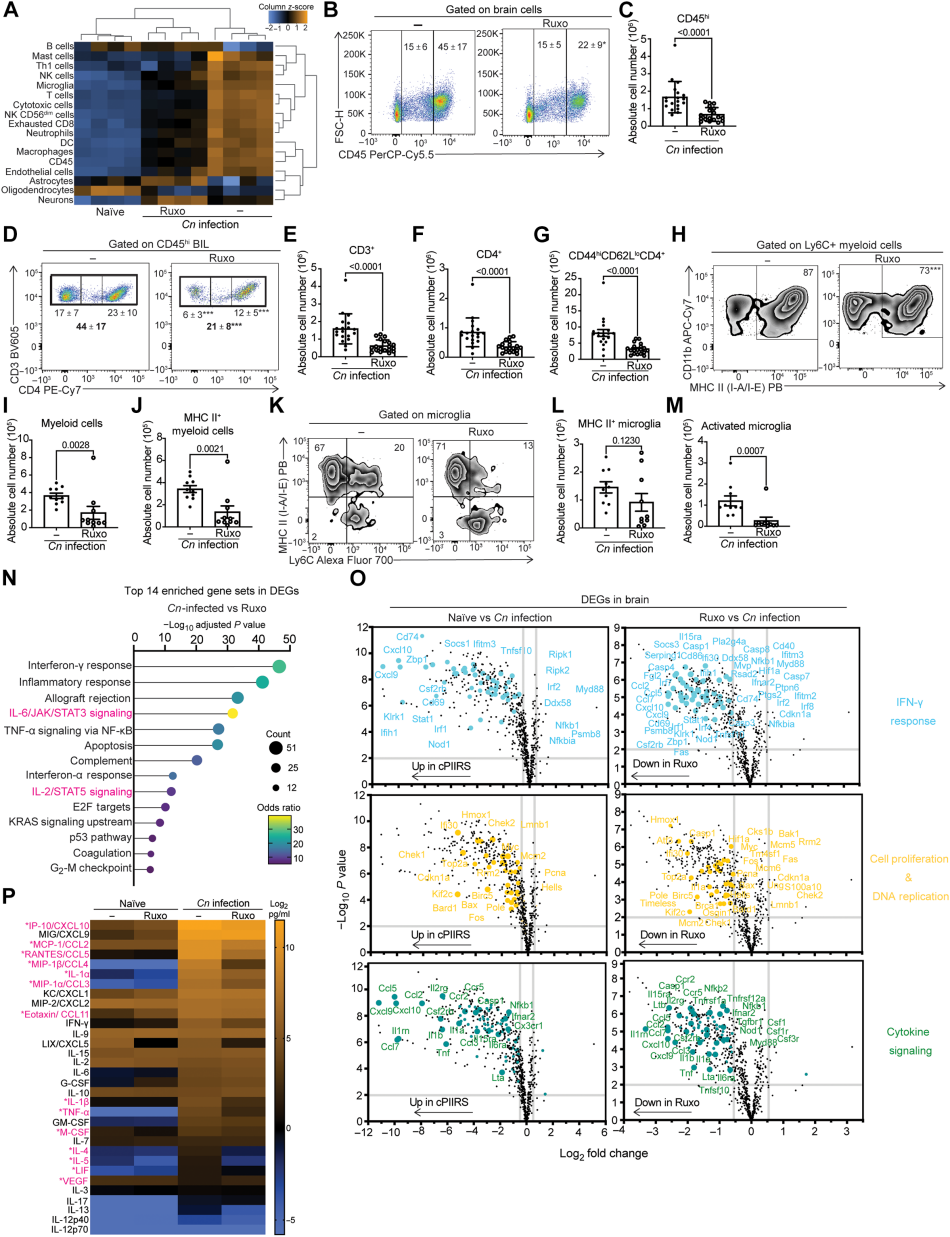
Ruxolitinib reduces accumulation of activated T cells and inflammatory myeloid cell populations and suppresses neuroinflammatory gene expression during murine cPIIRS. (**A**) At 21 dpi, predicted brain cell-type abundance was imputed from whole-brain mRNA obtained as in Fig. 2 (B to D) (*n* = 4 mice per group). (**B** to **M**) At 21 dpi, brains were harvested, brain-infiltrating lymphocytes (BIL) were isolated, and flow cytometry was performed to characterize live brain mononuclear cell populations. Representative flow plots with the proportion of indicated gated population (named above plot) and absolute number of brain cell population were enumerated for CD45^hi^ and CD45^int^ cells [(B) and (C)], CD3^+^ and CD4^+^ T cells [(D) to (F)], CD44^hi^CD62L^lo^ effector CD4 T cells (G), Ly6C^+^ myeloid cells expressing MHC II [(H) to (J)], microglia expressing MHC II and Ly6C [(K) and (L)], and MHC II^+^/Ly6C^+^ activated microglia [(K) and (M)]. Flow cytometry data shown are the means ± SD from two independent experiments with 10 to 20 mice per time point, and statistical significance was determined by unpaired Student’s *t* test, with *P* values indicated above each comparison. (**N**) Top 14 significantly enriched biological pathways identified in the brains of *Cn*-infected mice compared to ruxolitinib-treated/*Cn*-infected mice (MSigDB Hallmark 2020). JAK/STAT–specific pathways highlighted in pink. (**O**) Scatterplots comparing gene expression profiles in mouse brains. Highlighted are the IFN-γ response (top panels; light blue), cell proliferation and DNA replication (middle panels; yellow), and cytokine signaling pathways (bottom panels; green) (*n* = 4 mice per group). (**P**) Heatmap of cytokine and chemokine levels in whole-brain homogenates at 21 dpi. Data from two independent experiments with *n* = 6 mice per treatment group and arranged in order of the highest to lowest value for *Cn*-infected/untreated mice. The pink asterisk indicates statistical significance (*P* < 0.05) between infected groups.

To further investigate mechanisms of JAK1/JAK2–dependent brain pathology during cPIIRS, we profiled the cellular, transcriptional, and cytokine/chemokine milieu of mouse brains for changes in inflammatory parameters dependent on JAK/STAT signaling. These more detailed studies focused on mice not receiving AMB treatment because (i) AMB treatment has been shown to result in confounding immunological effects, including promoting innate immune activation, inflammation, and toxicity (*29*, *30*); (ii) patients are typically no longer on AMB during subsequent onset and treatment of cPIIRS (*8*); and (iii) AMB antifungal activity differs with fungal strains, reducing the general applicability of the results to clinical therapeutics (*31*). We first assessed global changes in predicted cell type abundance in whole brains at 21 dpi on the basis of NanoString Neuroinflammation gene expression data and cell profiling analysis (*23*, *32*). Compared to the brains of naïve mice, brains of mice with cPIIRS show increased transcriptional abundance of all immune cell types profiled, with a decreased abundance of CNS cell–associated expression (astrocytes, oligodendrocytes, and neurons) suggestive of neuronal depletion or injury (Fig. 4A). Ruxolitinib treatment increased the abundance of transcripts associated with astrocytes and neurons. To confirm increased inflammatory immune populations in the brain at 21 dpi, brain-infiltrating lymphocytes were isolated and flow cytometry was performed (gating strategy in fig. S4A), which demonstrated increased CD45^hi^ lymphocytes (Fig. 4, B and C), CD3^+^ T cells (Fig. 4, D and E), CD4^+^ T cells (Fig. 4F), and CD44^hi^CD62L^lo^ effector CD4 T cells (Fig. 4G) with cPIIRS and significant reductions to all populations after treatment with ruxolitinib (Fig. 4, B to G). Brain inflammatory myeloid populations were also reduced (Fig. 4, H to M) after ruxolitinib treatment, specifically inflammatory MHC II^+^ myeloid cells (Fig. 4, H to J) and activated microglia dually expressing Ly6C^+^ and MHC II^+^ (Fig. 4, K to M). These data confirmed the DEG-associated cellular data and suggest a role for an orally administered JAK inhibitor ruxolitinib in limiting the recruitment of inflammatory cells to the brain in a murine cPIIRS model.

We next used ruxolitinib-associated DEGs to provide an epistatic analysis of associated pathways driving cPIIRS. As expected, ruxolitinib-treated cPIIRS mouse brains showed reductions in IL-6/JAK/STAT3 signaling (Fig. 4N), with 44% of DEGs being JAK/STAT signaling dependent (Fig. 2, C and D, fig. S4B, and table S4). In addition, 55% of up-regulated DEGs in *Cn*-infected mouse brains compared to naïve mouse brains overlapped with DEGs down-regulated in ruxolitinib-treated/*Cn*-infected mice (Fig. 2, C and D). We found an enrichment of genes involved in the interferon-γ (IFN-γ) response pathway reduced with ruxolitinib treatment (Figs. 2E and 4, N and O, top panel), indicating that this biological process is prominently hypostatic to the JAK/STAT pathway and thus amenable to JAK inhibition. Further RNA profiling identified two biological processes that likely account for the reductions in inflammatory immune cells in the brain following ruxolitinib treatment: cell proliferation and DNA replication (E2F targets, G_2_-M checkpoint, p53 pathways; Fig. 4, N and O, middle panel) and cytokine/chemokine signaling (Fig. 4O, bottom panel). Closer examination of these cytokine/chemokine signaling DEGs (i.e., *Il1a*, *Il1b*, *Cxcl9*, *Cxcl10*, *Ccl5*, and *Ccl7*) indicated a *Cn* infection–specific up-regulation in inflammatory cell recruitment factors (Fig. 4O, bottom panel) that maintain immune cells within the brain*.* Ruxolitinib treatment significantly reduced these gene signatures and was confirmed by reduced protein levels including IFN-γ response–dependent CXCL10, CCL5, CCL3, IL-1α, and IL-1β proteins measured by Luminex in the brains of ruxolitinib-treated/*Cn*-infected mice (Fig. 4P). Together, these data indicate that ruxolitinib treatment interferes with cPIIRS-mediated neuroinflammation in two ways: reducing the activity potential of a broad array of cytokines necessary for immune cell survival and cPIIRS pathology and driving down-regulation of transcriptional signatures important for immune cell proliferation and activation within the brain.

### Ruxolitinib treatment in patients with cPIIRS was safe and effective and reduced CNS inflammation

On the basis of findings indicating hyperactive JAK/STAT signaling in the brains of patients with cPIIRS and cPIIRS mice and preclinical efficacy of ruxolitinib in mouse models, we explored whether ruxolitinib in FDA-approved dosages could reduce inflammation in seven courses of therapy of six patients with cPIIRS refractory to corticosteroid therapy (table S5). All patients presented to the National Institutes of Health (NIH) Clinical Center under an approved protocol (NCT00001352) following a diagnosis of CM and cPIIRS. Five of six patients were initially treated with pulse corticosteroid therapy as described (*8*) but continued to be symptomatic from cPIIRS after 1 to 6 months (table S6). Because of this prolonged requirement for corticosteroid therapy, FDA-approved doses of ruxolitinib (10 mg twice daily) were initiated, while corticosteroids and fluconazole were maintained at constant doses. Two patients (patients 5 and 6) were directly initiated on ruxolitinib, including one patient (patient 5) after a discussion of risks and benefits following a positive serological test for Chagas disease, which is a contraindication for corticosteroids (*33*).

**Fig. 5.**
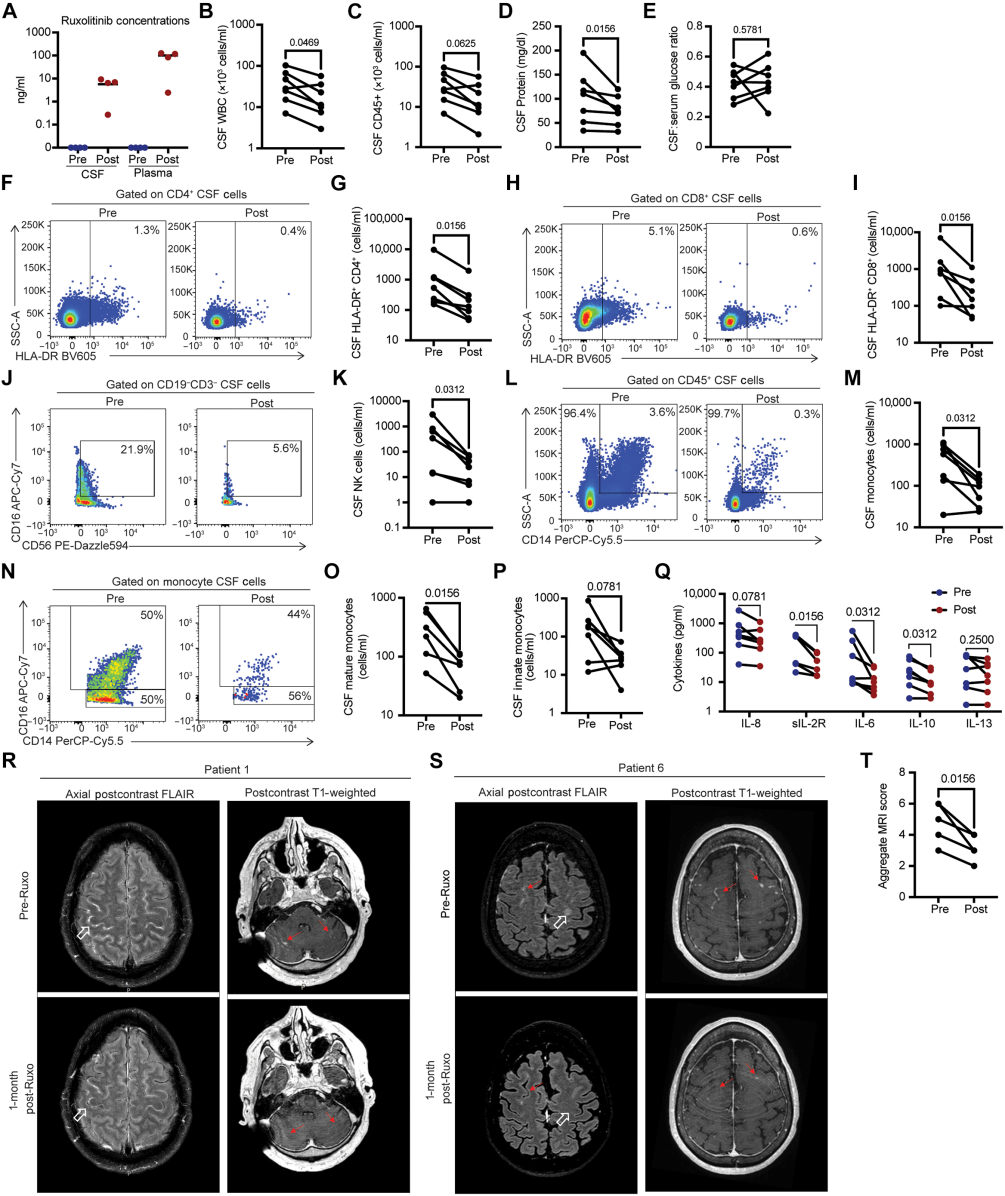
Ruxolitinib improves cellular and soluble markers of CSF inflammation and neurological findings in patients with cPIIRS. Lumbar punctures and brain MRI imaging studies were performed before ruxolitinib treatment (Pre) and at 1-month postruxolitinib treatment initiation (Post) in six patients, maintaining a constant dose of corticosteroids throughout ruxolitinib treatment (*n* = 6 patients with 7 treatment courses). (**A**) Concentrations of ruxolitinib in patient CSF and plasma. The line indicates the median value for four patients. (**B**) WBC counts, (**C**) CSF CD45+ cell counts, (**D**) CSF protein, and (**E**) glucose/serum ratio were assessed as described in Methods. (**F** to **P**) CSF T cell activation (HLA-DR+ expression) on CD4 [(F) and (G)] and CD8 T cells [(H) and (I)], CD56+ NK cells [(J) and (K)], and CD14+ monocytes [(L) and (M)], including both mature [(N) and (O)] and innate monocytes [(N) and (P)], was measured by flow cytometry at the baseline (postpulse, before ruxolitinib treatment) and 1 month following ruxolitinib initiation. Representative and summary data are shown. (**Q**) Concentrations of indicated soluble cytokines were measured from the CSF as described in Methods. (**R**) Patient 1: Axial postcontrast fluid-attenuated inversion recovery (FLAIR) images of the brain reveal meningeal enhancement observed in multiple convexity sulci at the baseline. Postruxolitinib, meningeal enhancement has decreased (open white arrow). Postcontrast T1-weighted images demonstrating abnormal enhancement (solid red arrow) along the cerebellar folia bilaterally, which resolved 1 month postruxolitinib. (**S**) Patient 6: Axial postcontrast FLAIR and postcontrast T1-weighted images of the brain reveal meningeal enhancement in convexity sulci (red arrows) along with edema in the adjacent cortical regions (open white arrow) at the baseline. Postruxolitinib, the meningeal enhancement improved with the resolution of edematous changes in the adjacent parenchyma (open white arrow). (**T**) Aggregate MRI scores (*n* = 7). CSF parameters and MRI scores analyzed using the Wilcoxon-matched pair signed-rank test, with *P* values indicated above each comparison.

After 1 month of ruxolitinib treatment (10 mg twice daily), all patients reported improvement in symptoms, with some regaining activities they were unable to perform on corticosteroid alone (Table 1). Ruxolitinib achieved a median plasma level of 99.9 ng/ml (*n* = 4 patients; Fig. 5A) and a median CSF level of 6.6 ng/ml (*n* = 4 patients; Fig. 5A). These plasma levels of ruxolitinib are similar to historic levels from patients on ruxolitinib and antiretroviral therapy and not taking fluconazole (*34*). Ruxolitinib treatment decreased CSF inflammatory markers including CSF white blood cell (WBC) count (Fig. 5B and fig. S5B) with a trend toward reductions in total CD45^+^ CSF cells (Fig. 5C), as well as CSF protein levels (Fig. 5D), but without changes in CSF glucose, which had normalized for most patients before the start of ruxolitinib treatment (Fig. 5E). Flow cytometry demonstrated statistically significant reductions in cellular markers of inflammation including CSF HLA-DR^+^ CD4^+^ (Fig. 5, F and G, and fig. S5C), HLA-DR^+^CD8^+^ (Fig. 5, H and I), and CD56^+^ NK cells (Fig. 5, J and K). A median 4.4 (±2.2 SD)–fold reduction of CSF HLA-DR^+^ CD4^+^ cells was observed, which has found utility as a surrogate of treatment response during cPIIRS with reductions in levels correlating with disease improvement (*8*). Myeloid CSF subset reductions included total CD14^+^ monocytes (Fig. 5, L and M, and fig. S5D) and mature monocytes (Fig. 5, N and O) with trends in reduction for innate monocytes (Fig. 5, N and P). Pro-inflammatory CSF cytokines and soluble markers were also reduced following ruxolitinib treatment, including the following: sIL-2R, a soluble marker of T cell activation (Fig. 5Q and fig. S5E), IL-6 (Fig. 5Q and fig. S5F), and IL-10 (Fig. 5Q and fig. S5G). IL-8 showed near-significant reduction and no major changes in the T helper 2 cytokine IL-13 (Fig. 5Q). No further reductions were seen in IFN-γ, IL-1β, TNF-α (tumor necrosis factor–α), IL-12, IL-17, IL-2, IL-4, and IL-5 levels in the CSF (fig. S5H).

**Table 1.** Efficacy of 1-month ruxolitinib treatment for steroid-refractory cPIIRS.

Patient no.	Baseline (preruxolitinib)	Postruxolitinib
1		
Course no. 1	headaches: 6/10	“minimal” headaches: 1/10
	tinnitus	
Course no. 2	headache: 7/10	markedly improved headaches at 1 month, intermittent at2 months, and no headaches at 2.5 months
2	minimally interactive	fully interactive, following commands
	bilateral lower extremity weakness	participates in physical therapy, able to stand with assistance
3	gait imbalance	no falls
	tremors	less dizziness
	moderate headaches	headaches resolved
4	decreased hearing (one side)	hearing stabilized
	MOCA: 22/30	feeling well
	headaches constant	headaches only intermittent
5	constant, moderate headaches: 4/10, accompanied by blurry vision, nausea/vomiting	headaches and all symptoms resolved (occasional <1/10)
	unable to work	resumed working as a truck driver
6	frequent headaches: 5/10	intermittent headaches: 3/10
	poor vision with optic atrophy	poor vision continues

Brain MRI with contrast was performed on all six patients before and ~1 month after initiation of ruxolitinib therapy. The most common radiological findings reported before initiation of ruxolitinib were meningeal enhancement (six patients), hydrocephalus or shunted ventricular system (six patients), nonenhancing basal ganglia lesions (three patients), and parenchymal enhancement (three patients; Fig. 5, R and S). Following ruxolitinib therapy, patients showed marked decreases in parenchymal and meningeal enhancement while on a constant dose of prednisone (Fig. 5, R and S) and all patients showed reductions in aggregate MRI scores defined previously (*8*) with a significant functional improvement overall postruxolitinib (Fig. 5T). In summary, patients who demonstrated persistent inflammation despite corticosteroid therapy demonstrated reduced intrathecal inflammation after ruxolitinib therapy.

All patients were monitored with weekly complete blood counts and liver function tests to evaluate for adverse events. All patients were continued on fluconazole while on immunosuppression, and no recurrence of CSF fungal growth or increases in cryptococcal blood antigens were noted. In addition, the patient with a positive serology for Chagas disease underwent an electrocardiogram and cardiac echo, which were normal, as well as monthly Chagas polymerase chain reactions, which remained negative. All patients tolerated ruxolitinib well without evident toxicity (table S7), except one patient who developed a fivefold increase in transaminase, which normalized with a reduction in ruxolitinib and abstinence from an episode of alcohol abuse. For the four patients on corticosteroids at the time of ruxolitinib initiation, all patients were able to successfully taper off prednisone while on ruxolitinib, although one patient (patient 2) was treated concurrently at the beginning with a parenteral pulse of corticosteroids before he eventually was tapered off. Because of subjective clinical improvements, some patients elected to continue ruxolitinib therapy with an overall mean duration of 4.4 months (±4.3-month SD; table S5). Two patients required continued immune suppression and were continued on ruxolitinib for up to 1 year. All patients are now off corticosteroids with symptomatic improvements in headaches and concentration sufficient to return to work (Table 1). One patient treated with ruxolitinib alone was also able to return to work since diagnosis after 1 week of therapy. Overall clinical benefit may be evident, but small numbers and lack of placebo controls make such a conclusion difficult.

In summary, these data suggest that ruxolitinib in FDA-approved dosages reduced CSF inflammation in this small cohort of patients with fungal cPIIRS and was well tolerated, supporting further study as a potential steroid-sparing agent in this disease. The data also suggest that CSF transcriptomics of immune pathology may be useful to accelerate the discovery of innovative therapeutics for neuroinflammatory diseases such as cPIIRS.

## DISCUSSION

cPIIRS is a devastating syndrome caused by excessive and aberrant immune responses in the brain and is responsible for considerable morbidity and mortality in CM for which limited treatment options exist. The present study adopted an approach to therapeutic selection for patients with cPIIRS termed pathway-instructed therapeutics, which identified JAK/STAT signaling prominence underlying this inflammatory disease through upstream analysis of transcriptional profiles of patient CSF cells—the first report, to our knowledge, of such nonbiased analysis in an infectious neuroinflammatory disease. The role of JAK/STAT signaling in promoting neuroinflammation was mechanistically probed using a cPIIRS mouse model and the JAK1/2 inhibitor ruxolitinib. Furthermore, we demonstrated that JAK/STAT-dependent signaling is necessary to drive hypercytokinemia (IFN-γ, CXCL10, CCL2, CCL5, CCL4, IL-1α, etc.) within the CNS and for persistence and replication of inflammatory and cytotoxic innate and adaptive immune cells at foci of fungal antigens in the brain causing debilitating neuronal damage, and all these processes are inhibited by ruxolitinib. Our encouraging preclinical data led to the use of ruxolitinib in seven courses of therapy for six patients who had continued CSF inflammation and suboptimal clinical response despite corticosteroid therapy. Ruxolitinib treatment significantly ameliorated the transcriptional, immunological, and MRI imaging readouts of brain pathology, which corresponded with patients with cPIIRS, subjectively noting clinical improvement in symptoms and quality of life. Two patients were able to return to work who were previously unable, and the short courses of ruxolitinib were well tolerated by all in this cohort, with one having modest reductions in hemoglobin that later improved without intervention. This study highlights a potential target for therapeutic intervention to be studied further in randomized clinical trials.

This study adds to our knowledge of biological mechanisms underlying the aberrant immune activation and neuropathology by fungal antigens with marked similarities between patients and the mouse model. scRNA-seq analysis and bulk RNA analysis of patient CSF revealed the breadth and magnitude of immune cells engaged by JAK/STATs during cPIIRS. Gene signatures of this pathway were involved in the recruitment and activation of pathogenic cytotoxic lymphocytes and activated monocytes (recruitment: *CCR2*, *CCL2*, *CCR5*, *CCL4*, etc.; cytokine production and response: *MAVS*, *NOD1*, *IL6R*, *KAT2A*, *RIPK1*, *IRF1*, *IRF3*, *XCL1*, *IL2RG*, *SYK*, etc.; activation: *CD86*, *CD74*, and *CD84*; cytotoxicity: *LAMP1*, *GNLY*, *PRF1*, *GZMB*, *NKG7*, etc.; innate responses: *CD74*, *FCER1G*, *CLEC7A*, *PTPRC*, *TREM2*, *DOCK2*, *CD300LF*, *FCGR2B*, *TNF*, etc.)—as well as a biological attempt by the immune system to respond to host damage caused by this immune pathology—and gene signatures were implicated in response to host damage [ATM-mediated DNA damage repair and oxidative stress response (*35*): *TOPBP1*, *CASP2*, *RAD50*, *APEX1*, *UNG*, *FEN1*, etc.]. Similarly, in examining the predominant transcriptional regulatory protein interaction networks in cPIIRS neuroinflammation, we identified significant cross-talk and co-regulation of the STAT1/STAT3 pathways with the AR (androgen receptor), TP53 (tumor suppressor p53), E2F1 (E2F transcription factor), and CTNNB1 (β-catenin) pathways in the CNS. The coordinated response of these regulators during neuroinflammation has not been reported but may shed insight into the complexity of cPIIRS and reveal future targets for modulation. Each has been implicated in different aspects of neuroinflammation affecting the CNS function. Specifically, AR is necessary for myelin regeneration and astrocyte function within the CNS (*36*), processes that require STAT3 signaling (*37*) and are dysregulated after traumatic brain injury (*38*, *39*). TP53 and E2FI have shown a cooperative role in mediating apoptosis (*40*) in response to oxidative stress in neurodegenerative diseases, such as Parkinson’s disease (PD) (*41*). Last, STAT1 is an upstream regulator of the Wnt/β-catenin pathway inhibiting repair functions (neuronal stem cell self-renewal, neurogenesis, and oligodendrogenesis) during neuroinflammation (*42*). As has been seen with PD and, more recently, with autoimmune polyendocrine syndrome type 1 (*26*), IFN-γ responses are hypostatic to these downstream JAK/STAT–dependent processes implicated in brain pathology (*43*). Most active viral meningitides are thought to be mediated by JAK/STAT signaling with many viruses acting against this pathway to suppress viral clearance, such as herpes simplex virus type 1, which suppresses the interferon signaling pathway via induction of the JAK/STAT suppressor, suppressor of cytokine signaling, SOCS3 (*44*). However, it is important to note that the biological processes and networks identified here in the CSF may not capture all the processes occurring within the brain parenchyma, where neuron damage is occurring.

cPIIRS has unique features compared to other CNS postinfectious inflammatory syndromes. HIV-associated *Mycobacterium tuberculosis*–immune reconstitution syndrome appears to be inflammasome, IL-8, and hypoxia-inducible factor–1α mediated with much smaller contributions from JAK signaling (*45*). Other postinfectious syndromes are believed to be driven more by specific autoantibodies such as the movement disorder, anti-*N*-methyl-d-aspartate receptor encephalitis targeting the *N*-methyl-d-aspartate receptor (*46*), or acute disseminated encephalitis, a multiple sclerosis–like syndrome characterized by widespread inflammation in the brain and spinal cord following a viral or bacterial infection accompanied by perivenous demyelination linked to antibodies to native myelin oligodendrocyte glycoprotein (*47*). However, a role for IL-6 signaling correlating with these autoantibodies could suggest an additional role for JAK/STAT as IL-6 signals through this pathway (*15*, *47*), suggesting a need for discriminatory pathway analysis in neurologically related infections.

Since its FDA approval in 2011 for the treatment of myelofibrosis (*48*), the clinical use of ruxolitinib as a selective JAK1 and JAK2 inhibitor has greatly expanded (*49*–*58*). However, several dozen anti-inflammatory agents have been FDA approved in the past 10 years directed against diverse targets and pathways, including PDE4 (apremilast), S1P receptor (ozanimod), and a variety of cytokines including IL-17A (secukinumab), IL-23 (guselkumab), and integrins (vedolizumab), making target selection a challenge. Such a large armamentarium makes directed therapy more facile but also presents difficulties in repurposing such agents based on hypothesis-based testing alone. For example, investigating pathways in hemophagocytic lymphohistiocytosis has required the investigation of at least 40 different pathways in seminal work spanning several decades (*59*). In contrast, nonbiased approaches such as the pathway-instructed therapeutic approach demonstrated here offer the promise to accelerate product development including repurposing studies demonstrated herein. These results have led to the design of a randomized controlled trial of ruxolitinib in the treatment of cPIIRS at the NIH Clinical Center. We do not suggest the treatment of ruxolitinib for cPIIRS outside a study protocol at present.

A limitation to this study is that it represents a small cohort study that may suffer from referral bias of severe cases to the NIH from outside hospitals and is not sufficiently powered or controlled to determine the efficacy of ruxolitinib for the treatment of cPIIRS. Thus, the goal is not to make definitive conclusions of immunosuppressive complications or toxicities. Instead, we highlight the need for randomized studies to determine whether the clinical efficacy of steroids can be potentiated by ruxolitinib and/or whether it may benefit long-term outcomes and reduce corticosteroid requirements in cPIIRS. It is also not known whether inhibition of the JAK/STAT signaling pathway through the use of a specific JAK1 inhibitor would be as efficacious from the treatment of cPIIRS and whether JAK/STAT signaling inhibition could be efficacious for HIV-related cIRIS.

In summary, we identify a prominent role for JAK/STAT signaling in a fungal inflammatory disease. Furthermore, pathway-instructed therapeutics used identified ruxolitinib treatment for steroid-refractory cPIIRS, in which six patients had measured and meaningful reductions in biomarkers of CNS inflammation while maintaining a constant dose of corticosteroids. All patients were able to taper completely off corticosteroids, and at this time, all patients are clinically stable with continued negative fungal cultures after completion of immunosuppressant therapy.

## MATERIALS AND METHODS

### Ethics statement

The National Institute of Allergy and Infectious Diseases (NIAID) Institutional Review Board approved this study under NIAID protocol number 93-I-0106. All subjects provided written informed consent directly or via their durable power of attorney.

### Study design and participants

All six patients were seen at the NIH Clinical Center, Bethesda, MD, between 2020 and 2024 as part of a prospective observational study examining host genetics and immunology of cryptococcal disease in previously healthy, non–HIV-infected adults. Patients were excluded if they were <18 years old and had any underlying immune deficiency, such as HIV or receipt of immunosuppressant medications, including cancer chemotherapy or monoclonal antibodies. A diagnosis of CM was defined as a positive latex agglutination cryptococcal antigen and/or the isolation of *Cryptococcus* in one or more CSF cultures. The six patients analyzed for the present study had completed standard antifungal therapy of liposomal AMB and flucytosine for at least 2 weeks, followed by fluconazole at another institution before NIH transfer, and had negative CSF fungal cultures. All patients underwent referral to the NIH after they developed refractory symptoms consistent with cPIIRS, including declining mental status and cranial nerve defects, and subsequently met criteria for cPIIRS.

### Protocol

At the NIH, all patients were diagnosed with cPIIRS (*8*) and five patients (P1 to P4 and P6) received a pulse of 1 g of methylprednisolone parenterally daily for 7 days followed by prednisone (1 mg/kg per day) for 1 month followed by a taper as tolerated. Lumbar punctures were performed on admission, after a pulse of methylprednisolone, and as clinically required based on CSF opening pressures and symptoms. Ruxolitinib was used in patients poorly responsive to corticosteroid therapy, and data were collected retrospectively. Before treatment with ruxolitinib and to minimize the risk of ruxolitinib-associated infections, patients were screened for latent tuberculosis, viral hepatitis, herpes simplex virus 1 and herpes simplex virus 2, Epstein-Barr virus, cytomegalovirus, and causes of bacterial encephalitis. Patients were maintained on prophylactic antibiotics (fluconazole and sulfamethoxazole/trimethoprim) and antiviral agents (acyclovir) to prevent reactivation. Spinal fluid was sent for routine analysis, fungal cultures, cryptococcal antigen titers, immunophenotyping, soluble cytokine analysis as previously described (*4*, *60*), and CSF fluid component (glucose and protein) measurements. Retrospective analyses of CSF studies were reviewed to generate data for these studies. Neuroimaging of the brain via MRI was performed throughout treatment, as previously described (*61*). Radiological findings were scored on the basis of the presence of nine criteria; details of scoring can be found in (*8*). Aggregate scores were calculated for each patient ranging from a minimum of 0 to a maximum of 12. A decrease in aggregate scores on postruxolitinib MRI compared to the baseline indicated radiological improvement.

### CSF and PBMC collection, processing, and immunophenotyping by flow cytometry

CSF was collected on ice and processed immediately. A portion of the collected CSF (10 to 20 ml) was collected and assigned prospective alphanumeric codes. To determine whether viable or viable but not culturable *Cryptococcus* cells were present in patient CSF samples, 100 μl of CSF was plated on yeast extract, peptone, and dextrose (YPD) media or minimal media containing 125 μM pantothenic acid (reactivation media) (*62*, *63*), respectively, and grown at 37°C and 5% CO_2_ for at least 1 month. No patient CSF samples grew *Cryptococcus* on these media at any time throughout their treatment at the NIH. The CSF volume and visual appearance were recorded, and CSF samples were centrifuged at 300*g* for 10 min at 4°C within 15 min of collection. The CSF supernatant was aliquoted and stored in polypropylene tubes at −80°C and was tested for soluble cytokines by ARUP Laboratories (Salt Lake City, UT) by a method previously described (*4*). Cell pellets were resuspended in 500 μl of phosphate-buffered saline (PBS) containing 1% bovine serum albumin (BSA) and counted using a Luna-fl Dual Fluorescence Cell Counter (Logos Biosystems), and the number of WBCs per milliliter of CSF was calculated. A minimum of 5 × 10^4^ viable CSF cells were analyzed immediately by nine-color flow cytometry to enumerate absolute numbers of 10 subsets of CSF immune cells. A minimum of 2 × 10^5^ CSF cells were spun down and resuspended in 1 ml of RNAlater (Millipore Sigma, R0901) and stored at −80°C for NanoString analysis. Peripheral blood mononuclear cell (PBMC) isolation from whole blood was performed by density gradient centrifugation using Ficoll-Paque PLUS Premium (GE Healthcare, 17-5442-03) and SEPMate-50 tubes (STEMCell, 85450).

To assay for immune cell populations within the CSF and peripheral blood, CSF cells and PBMCs were first blocked with Fc Receptor Binding Inhibitor (Thermo Fisher Scientific, cat. no. 14-9161-73, RRID:AB_468582) in flow staining buffer (PBS containing 1% BSA) for 15 min at 4°C and then stained with antibodies to CD45 FITC (fluorescein isothiocyanate; HI30, Thermo Fisher Scientific, cat. no. 11-0459-42, RRID:AB_10852703), CD3 PB (UCHT1, BioLegend, cat. no. 300417, RRID:AB_493094), CD4 PE-Cy7 (SK3, BD Biosciences, cat. no. 348789, RRID:AB_400379), CD8 APC (SK1, BioLegend, cat. no. 344722, RRID:AB_2075388), HLA-DR BV605 (LN3, Thermo Fisher Scientific, cat. no. 406-9956-41, RRID:AB_3074085), CD19 Alexa Fluor 700 (HIB19, BioLegend, cat. no. 302226, RRID:AB_493751), CD59 (NCAM) PE/Dazzle594 (HCD56, BioLegend, cat. no. 392410, RRID:AB_2728406), CD14 PerCP-Cy5.5 (63D3, BioLegend, cat. no. 367109, RRID:AB_2566711), and CD16 APC-H7 (3G8, BD Biosciences cat. no. 560195, RRID:AB_1645466) for 30 min at 4°C. After incubation, cells were washed twice with flow staining buffer and fixed in Cytofix buffer (BD) for 30 min before washing. Data were immediately collected on an LSRFortessa flow cytometer (BD) with FACSDIVA software (BD) and analyzed using FlowJo software (BD). Appropriate unstained, single-color control and fluorescence minus one controls were run with samples.

All events were first gated using time versus FSC-H to exclude events at the start and end of the run, which often have altered light scattering profiles. Next, debris and cell aggregates were excluded using FSC-H versus FSC-W (gated for FSC singlets) and SSC-H versus SSC-W (gated for SSC singlets) followed by gating on CD45+ CSF cells (SSC-H versus CD45) to exclude nonleukocytes. Activated CD4+ T cells were defined as CD45^+^CD14^−^CD3^+^CD19^−^CD4^+^HLA-DR^+^. Activated CD8+ T cells were defined as CD45^+^CD14^−^CD3^+^CD19^−^CD8^+^HLA-DR^+^. Classical (mature) monocytes were defined as CD45^+^CD14^+^CD16^−^HLA-DR^+/−^ (*64*). Nonclassical (innate) monocytes were defined as CD45^+^CD14^+^CD16^+^HLA-DR^+/−^ (*64*). Unstained and fluorescence minus one control samples were used to define negative and positive populations for gating.

### Mice

All animal experiments were conducted under protocols approved by the Institutional Animal Care Committee of the Intramural NIH/NIAID and were performed in accordance with NIH guidelines and the Guide for the Care and Use of Laboratory Animals under protocol LCIM 12E (PHS assurance no. D16-00602). C57BL/6 female mice were obtained from Taconic Biosciences Inc. (B6NTac, RRID:IMSR_TAC:B6, Germantown, NY). Mice were housed under specific pathogen–free conditions in microisolator cages at the NIH and were provided with food and water ad libitum. Mice were 6 to 12 weeks old at the time of infection, were randomized to infection and treatment with ruxolitinib and antifungal treatment, and were humanely euthanized by isoflurane inhalation at the time of data collection. For mortality studies, mice were euthanized when they lost 20% body weight, had persistent cranial swelling, and/or developed neurological symptoms.

### Ruxolitinib treatment

Ruxolitinib (R-6688, LC Laboratories) was formulated into Nutragel diet (F5769-Kit, Bio-Serv) at a concentration of 1 g/kg, as previously described (*25*, *26*). Mice were acclimated to untreated Nutragel diet (replaced daily) alongside normal rodent chow for 7 days. Mice were provided with ruxolitinib diet or Nutragel diet ad libitum (replaced daily) for 2 days before infection, which continued throughout the infection study period. For dose calculation of the mouse daily intake of ruxolitinib, mice were housed in groups of five and fed from a 57-g Nutragel cup per day. Mice typically consumed ~60 to 75% of this diet per day, with each mouse consuming 6.8 to 8.5 g of Nutragel diet per day or 6.8 to 8.5 mg of ruxolitinib per mouse per day. This is consistent with therapeutic doses delivered by subcutaneous injection by others (*65*). In experiments where mice were treated with AMB during infection, ruxolitinib diet was added on day 18 postinfection and continued throughout the study period.

### AMB treatment

AMB (Sigma-Aldrich, A9528) was dissolved in sterile pharmaceutical-grade saline at 1 mg/ml. Mice received a daily dose of AMB (5 mg/kg) intraperitoneally beginning on day 14 postinfection, which continued throughout the study period.

### *Cryptococcus neoformans* and cPIIRS model mouse infection

ATCC 24067 (American Type Culture Collection, Manassas, VA), *Cryptococcus neoformans* 52D strain, was used to infect mice in this study. Cryptococcal cells from frozen stocks (10% glycerol) were plated on YPD (BD Difco) agar plates containing chloramphenicol (Sigma-Aldrich) at 30°C. Single colonies were subsequently picked and grown overnight in YPD broth (BD Difco) containing chloramphenicol (50 mg/ml) at 30°C with 225-rpm shaking. Fungal cells were washed three times in PBS, counted on a hemocytometer with trypan blue, and adjusted to a concentration of 5 × 10^6^/ml before infection. Mice were infected with 10^6^ yeast cells (in 200 μl of sterile PBS) via tail-vein intravenous injection. Serial dilutions of the *C. neoformans* suspension were plated on YPD agar to confirm the number of viable fungi in the inoculum. Weights were monitored three times a week. For the analysis of brain fungal burdens, animals were euthanized, organs were harvested and weighed, and brains were homogenized in PBS and serially diluted before plating onto YPD agar supplemented with chloramphenicol (Sigma-Aldrich). Colonies were counted after incubation at 30°C for 48 hours. For the analysis of brain cytokines, brain homogenates were diluted twofold in PBS before measurement using the Mouse Cytokine 32-plex Discovery Assay (Mouse Cytokine Array/Chemokine Array 32-Plex Panel; cat. no. MD31; Eve Technologies, Alberta, Canada).

### Analysis of murine cells by flow cytometry

Leukocytes in the brain were isolated as previously described (*16*). Briefly, mice were euthanized and perfused with 10 ml of PBS to remove circulating red blood cells and leukocytes from the brain. The brains were aseptically removed, transferred to gentleMACS C tubes containing 5 ml of sterile complete RPMI 1640 [with 5% fetal bovine serum, 25 mM Hepes, GlutaMAX, penicillin-streptomycin, and enzyme cocktail containing collagenase (50 μg/ml; Roche), hyaluronidase (Roche), and deoxyribonuclease (100 U/ml; Roche)]. The tissue was processed on a gentleMACS homogenizer (Miltenyi Biotec) and incubated at 37°C for 45 min. Small samples of the homogenate were collected immediately after processing for whole-brain fungal burden, RNA, and cytokine measurements. The remaining homogenate was washed with RPMI 1640 and filtered through a 70-μm cell strainer. A 33/67% Percoll (GE Healthcare)/RPMI medium gradient was used to remove cell debris, myelin, and neurons, and then microglia and brain-infiltrating leukocytes were recovered from the pellet. Suspensions were centrifuged at 800*g* for 30 min at 4°C with the brake off. Isolated cells were washed twice with RPMI 1640 (with 5% fetal bovine serum and penicillin-streptomycin) to remove residual Percoll before use in assays. Total cell counts were obtained by counting live cells using a Luna cell counter.

For splenocyte isolation, spleens were removed and then mechanically dispersed by using a 3-ml sterile syringe plunger to press through a 70-μm cell strainer (BD Falcon, Bedford, MA) in complete RPMI 1640 medium. The cell suspension was washed with PBS and centrifuged. Erythrocytes in the cell pellets were lysed by the addition of 5 ml of NH_4_Cl buffer [0.829% NH_4_Cl, 0.1% KHCO_3_, and 0.0372% Na_2_EDTA (pH 7.4)] for 5 min, followed by addition of a 10-fold excess of RPMI 1640 medium. After centrifugation, the splenocytes were saved for further use.

Cells were stained with fixable LIVE/DEAD dye (Life Technologies), blocked with anti-CD16/32 (Thermo Fisher Scientific, cat. no. 14-0161-86, RRID:AB_467135), and stained with antibodies for CD45 (30-F11; BioLegend, cat. no. 103131, RRID:AB_893344), CD3 (17A2; BioLegend, cat. no. 100237, RRID:AB_2562039), CD4 (GK1.5; Thermo Fisher Scientific, cat. no. 25-0041-82, RRID:AB_469576), CD11b (M1/70; BioLegend, cat. no. 101226, RRID:AB_830642), Ly6C (HK1.4; BioLegend, cat. no. 128024, RRID:AB_10643270), MHC class II (M5/114.15.2; Thermo Fisher Scientific, cat. no. 48-5321-82, RRID:AB_1272204), CD44 (IM7; BioLegend, cat. no. 103008, RRID:AB_312959), and CD62L (MEL-14; Thermo Fisher Scientific, cat. no. 17-0621-82, RRID:AB_469410). Fluorescence minus one controls, single-color controls, and an unstained control were used for all experiments. Data were collected on an LSRFortessa (BD) and were analyzed using FlowJo (BD). Microglia are defined as CD45^int^CD11b^+^, while inflammatory monocytes are CD45^hi^CD11b^+^Ly6c^+^.

### NanoString RNA profiling and normalization analysis

Total RNA was isolated from mouse whole-brain homogenate using TRIzol (Life Technologies)/RNeasy Mini Kit (Qiagen), and gene expression was analyzed using the NanoString nCounter Mouse Neuroinflammation Panel (NanoString Technologies). Total RNA was isolated from human patient CSF cells (from four different patients with cPIIRS at the time of cPIIRS diagnosis and before PULSE corticosteroid treatment and from three non-PIIRS donors) stored in RNAlater (−80°C) gene expression analyzed using the NanoString nCounter Human Neuroinflammation Panel (NanoString Technologies). Human CSF samples were first lysed using buffer RLT [4 volume of buffer RLT:1 volume of CSF cells (in RNAlater)] and QIAShredder Kit (Qiagen). Next, RNA was isolated using the RNeasy Micro Kit (Qiagen) with on-column deoxyribonuclease treatment. RNA quality was assessed using an Agilent 2100 Bioanalyzer Expert and the Eukaryote Total RNA Pico Kit (Agilent Technologies). Ribosomal RNA peaks were visible in most of the samples analyzed. Because of the low concentration of RNA in CSF samples, 500 pg of total RNA was first processed using the nCounter Low RNA Input Kit with nCounter Human Neuroinflammation panel specific primers (NanoString). In addition, every sample was spiked in with the positive and negative controls provided with the panel. This technique uses capture and reporter probes specific for each gene labeled with a unique fluorescent barcode. Signals generated from these barcodes are automatically detected and quantified by the nCounter Pro Analysis System. The generated data were compiled as resource compiler files.

Resource compiler files were imported into nSolver 4.0 analysis software (NanoString), and quality control steps were performed following the manufacturer’s guidelines. Raw data were processed sequentially: First, background subtraction was performed using the following formula: Read count of each gene − 2 × (Mean + 2 × SD) of all negative controls. Next, the positive control normalization factor was calculated according to the manufacturer’s instructions using positive controls that were spiked into every sample. Then, the CodeSet content normalization factor (housekeeping normalization factor) was calculated using all reference genes to adjust for differences in analyte abundance and/or analyte quality across samples. The most stably expressed housekeeping genes (10 of 14 for human data and 7 of 14 for mouse data) were selected using the geNorm algorithm (*66*). Normalization was performed per the default settings. The selection criteria for the positive control normalization factor were 0.3 to 3.0. Pathway scores were calculated as the first principal component of the pathway genes’ normalized expression. The software will orient the scores such that an increased score corresponds with increased expression in most pathway genes. Scores of the cell-type characteristic genes were analyzed using the default settings from the cell-type profiling algorithm in the nSolver analysis software.

### Bioinformatic analysis

To identify the biological processes represented by the expressed genes, upstream regulators, and upstream regulator interactions needed to illicit the changes in expression that we see in the genes, the IPA platform (Qiagen) and Enrichr Analysis were used (*67*–*69*). The thresholds of *P* < 0.05 and log_2_ fold change > 0.58 were used to obtain significant DEGs (Figs. 1, B to F, 2, C to G, and 4, N and O). The ChEA Transcription Factor Targets STAT1 and STAT3 datasets were used to identify STAT1- and STAT3-dependent genes from these DEGs (*70*). Gene set enrichment analysis was performed via imputation of DEGs using the Molecular Signatures Database (MSigDB) Hallmark gene set (*14*, *71*). Pathway analysis using DEGs was performed using Enrichr TRRUST Transcription Factors 2019 Database and the Enrichr Transcription Factor PPI to construct networks. Displayed in figures (Fig. 1, E and F) are the top 10 regulators identified on the basis of adjusted −log_10_ *P* value and graphed according to the percent of pathway-specific genes from human datasets identified in the DEGs. The fraction next to each bar shows the number of DEGs identified out of total number of genes regulated by each pathway according to the database.

### Generation of single-cell CSF libraries and sequencing

Single Cell 3′ version 3 gene expression libraries were sequenced on a NextSeq 2000 P3 flow cell at a 28-cycle + 90-cycle asymmetric run. 10x Genomics Cell Ranger version 7.2.0 was used to generate FASTQ files and count matrices for analysis using default parameters with the GRCh38 transcriptome reference (GRCh38-2024-A). Seurat version 5.0.3 (*72*) was used to filter out cells with a gene count less than 500 or greater than 7500 and a mitochondrial gene expression greater than 10%. The log_10_ normalized data were visualized using uniform manifold approximation and projections (UMAP) with dimension reduction using 30 principal components. Unsupervised clustering was performed using a resolution of 2 and subclustering with a resolution of 0.2. Cell clusters were annotated using Azimuth PBMC (*73*) and adipose (*74*) cell-type specific markers in conjunction with Schafflick *et al.*’s paper (*75*) and differential expression performed using MAST (*76*). Single-cell plots were created using the scCustomize package (*77*).

### Western blot analysis

Western blotting using indicated anti-mouse target antibodies was performed. For western blotting, brains were homogenized in Pierce RIPA Buffer (Thermo Fisher Scientific, 89900) with the addition of Halt Protease and Phosphatase Inhibitor Single-Use Cocktail (100×) (Thermo Fisher Scientific, 78442) and protein concentrations were determined using a colorimetric assay (Bio-Rad, 5000006). Eighty micrograms of each sample was subjected to 12% Mini-PROTEAN TGX Precast Protein Gels (10-well, 50 μl; Bio-Rad, 4561043), and proteins were transferred onto nitrocellulose membranes (GenScript, L00224). Membranes were immunoblotted with rabbit anti-phospho-Stat1 (Tyr^701^) (Cell Signaling Technology, cat. no. 9167, RRID:AB_561284), rabbit anti-Stat1 (Cell Signaling Technology, cat. no. 14994, RRID:AB_2737027), rabbit anti-phospho-Stat3 (Tyr^705^) (Cell Signaling Technology, cat. no. 9145, RRID:AB_2491009), rabbit anti-Stat3 (Cell Signaling Technology, cat. no. 4904, RRID:AB_331269), and rabbit anti-β-actin (Cell Signaling Technology, cat. no. 4970, RRID:AB_2223172). Membranes were then probed with Goat anti-Rabbit IgG (H + L) Cross-Adsorbed Secondary Antibody, HRP (Thermo Fisher Scientific, cat. no. G-21234, RRID:AB_2536530), and proteins were visualized using Clarity Western ECL Substrate (Bio-Rad, 170-5061).

### Immunofluorescence microscopy

The brains were fixed with 4% paraformaldehyde, cryoprotected in 30% sucrose for 72 hours, then frozen, and embedded in the optimal cutting temperature (OCT) compound. Antigen retrieval was carried out in citrate buffer in a boiling water bath for 15 min. Sections were blocked in 5% BSA in PBS. For the β-III tubulin and Syt-7 staining, sections were stained with a rabbit polyclonal β-III tubulin antibody (1:100, TuJ1, Abcam, cat. no. ab18207, RRID:AB_444319) and Syt-7 antibody (clone S275, Thermo Fisher Scientific, cat. no. MA5-27654, RRID:AB_2735383) for 1 hour at 37°C. Secondary antibody staining was done with goat anti-mouse Alexa 555 antibody (Thermo Fisher Scientific, cat. no. A28180, RRID:AB_2536164) and goat anti-rabbit Alexa 488 antibody (1:500, Thermo Fisher Scientific, cat. no. A32731, RRID:AB_2633280) for 45 min at 37°C. For the β-III tubulin and cleaved caspase-3 staining, sections were then stained with a primary antibody to β-III tubulin, a rabbit antibody specific to cleaved caspase-3 (1:100, Cell Signaling Technology, cat. no. 9664, RRID:AB_2070042), and a rat anti-mouse CD45 (clone 30-F11, BioLegend, cat. no. 103112, RRID:AB_312977) for 1 hour at 37°C. Sections were then washed in PBS and counterstained with the following fluorophore-conjugated secondary antibodies: goat anti-rabbit Alexa 555, goat anti-mouse Alexa 488, and donkey anti-rat Alexa 647 antibody (1:500, Thermo Fisher Scientific, cat. no. A78947, RRID:AB_2910635) for 45 min at 37°C. After that, the sections were washed in PBS for 30 min. The autofluorescence was quenched by Vector’s true view kit (Vector Laboratories) as per the manufacturer’s protocol before mounting with a mounting medium. Samples were visualized using a KEYENCE microscope BZ-X800 series. Images were captured by the camera provided by the vendor by using a ×20 objective, 0.45–numerical aperture Nikon. Images were deconvoluted for enhancing the clarity of the images. All the images were captured under the same exposure conditions. Grayscale images were pseudocolored for presentation. Mean fluorescence intensity (MFI) measurements were made on a linear scale. Lesion diameters were measured using the following formula: (Long axis + Short axis)/2 or (*L* + *W*)/2. Images were analyzed by ImageJ, and the numbers of damaged neurons were calculated by counting the numbers of cleaved caspase-3–positive neurons around the area infected by *Cn*. Areas that are 500 μm^2^ around the cryptococcal lesions were analyzed.

We used linear regression models fit via generalized estimating equations (GEE) to assess the effect of ruxolitinib, with and without AMB, on each of the outcomes. In each of the GEE models, we used an exchangeable working correlation structure and clustered on mouse ID. Lesion diameter and MFI measurements were modeled on the natural log scale with an identity link; hence, the exponentiated model coefficients are interpreted in terms of multiplicative changes on the geometric mean outcome. CD45 counts were modeled using a Poisson GEE with loglink. Models were fit using the geepack package in R (*78*). Given the exploratory nature of this study, no adjustment for multiple comparisons is made.

### Quantification of ruxolitinib by liquid chromatography–tandem mass spectrometry in plasma, CSF, and tissue

To measure levels in serum and brains of mice (at 21 dpi), whole blood was collected and placed in serum separator tubes (Sarstedt, 411378005) immediately, followed by extraction of brains. Brains were snap frozen and stored at −80°C. Brains were homogenized in 2 ml of methanol. To measure levels in mouse serum (at 21 dpi), human plasma (at the baseline and at 1 month on ruxolitinib), or human CSF (at the baseline and at 1 month on ruxolitinib), 50 μl of the sample was extracted with 250 μl of methanol. [^2^H_9_]-ruxolitinib dissolved in 50% methanol at 500 nM was spiked in serum/plasma/CSF (10 μl) or tissue samples (40 μl) as an internal standard before extraction. Supernatants were mixed (1:1) with water and then subjected to liquid chromatography–tandem mass spectrometry analysis. A Thermo TSQ Quantiva triple quadrupole mass spectrometer (Thermo Fisher Scientific, Waltham, MA) with an electrospray ionization interface was used for liquid chromatography–mass spectrometry analysis. Analytes were separated by a Kinetex XB-C8 column (50 by 2.1 mm, 2.6 μm; Phenomenex, Torrance, CA). Gradient elution was used for the separation with mobile phase A (0.1% formic acid) and mobile phase B (acetonitrile). The liquid chromatography gradient started with 10% of mobile phase B for 0.5 min, which then increased from 10 to 90% in 3 min and kept at 90% for 0.5 min before returning to the initial condition. Selected reaction monitoring in positive mode was used to detect ruxolitinib (307.1 → 186.1) and the internal standard [^2^H_9_]-ruxolitinib (316.2 → 186.1). Data were collected and processed by Thermo Xcalibur 3.0 software. Calibration curves were generated from standard ruxolitinib by serial dilutions in blank biometric samples (CSF/plasma: 1 nM to 2 μM; brain homogenates: 0.1 to 100 nM) using the same extraction method described above. The calibration curves had an *r*^2^ value greater than 0.99.

All the chemicals are of analytical grade or higher and were obtained commercially from Sigma-Aldrich (St. Louis, MO). [^2^H_9_]-ruxolitinib was purchased from ALSACHIM (lllkirch, Alsace, France) with purity greater than 98%.

### Data preparation

Figures were prepared using Adobe Illustrator (Adobe) and BioRender.

### Statistical analysis

Statistical analysis and graphical visualizations were performed using GraphPad Prism version 10 software, with statistical test performed detailed in each figure legend.

## Supplementary Materials

### This PDF file includes:

Figs. S1 to S5 Tables S1 to S7 References
