# Supplementary material for: Pathway-instructed therapeutic selection of ruxolitinib reduces neuroinflammation in fungal postinfectious inflammatory syndrome
Source: Sci Adv. 2025 Mar 21;11(12):eadi9885. doi: 10.1126/sciadv.adi9885 (PMC11927619; doi:10.1126/sciadv.adi9885)
Supplement: Supplementary file 1 — Figs. S1 to S5 Tables S1 to S7 References [file sciadv.adi9885_sm.pdf]

Science Advances
AAAS

Supplementary Materials for

Pathway-instructed therapeutic selection of ruxolitinib reduces neuroinflammation in fungal postinfectious inflammatory syndrome

Jessica C. Hargarten *et al.*

Corresponding author: Peter R. Williamson, williamsonpr@mail.nih.gov

Sci. Adv. **11**, eadi9885 (2025)
DOI: 10.1126/sciadv.adi9885

This PDF file includes:

Figs. S1 to S5
Tables S1 to S7
References

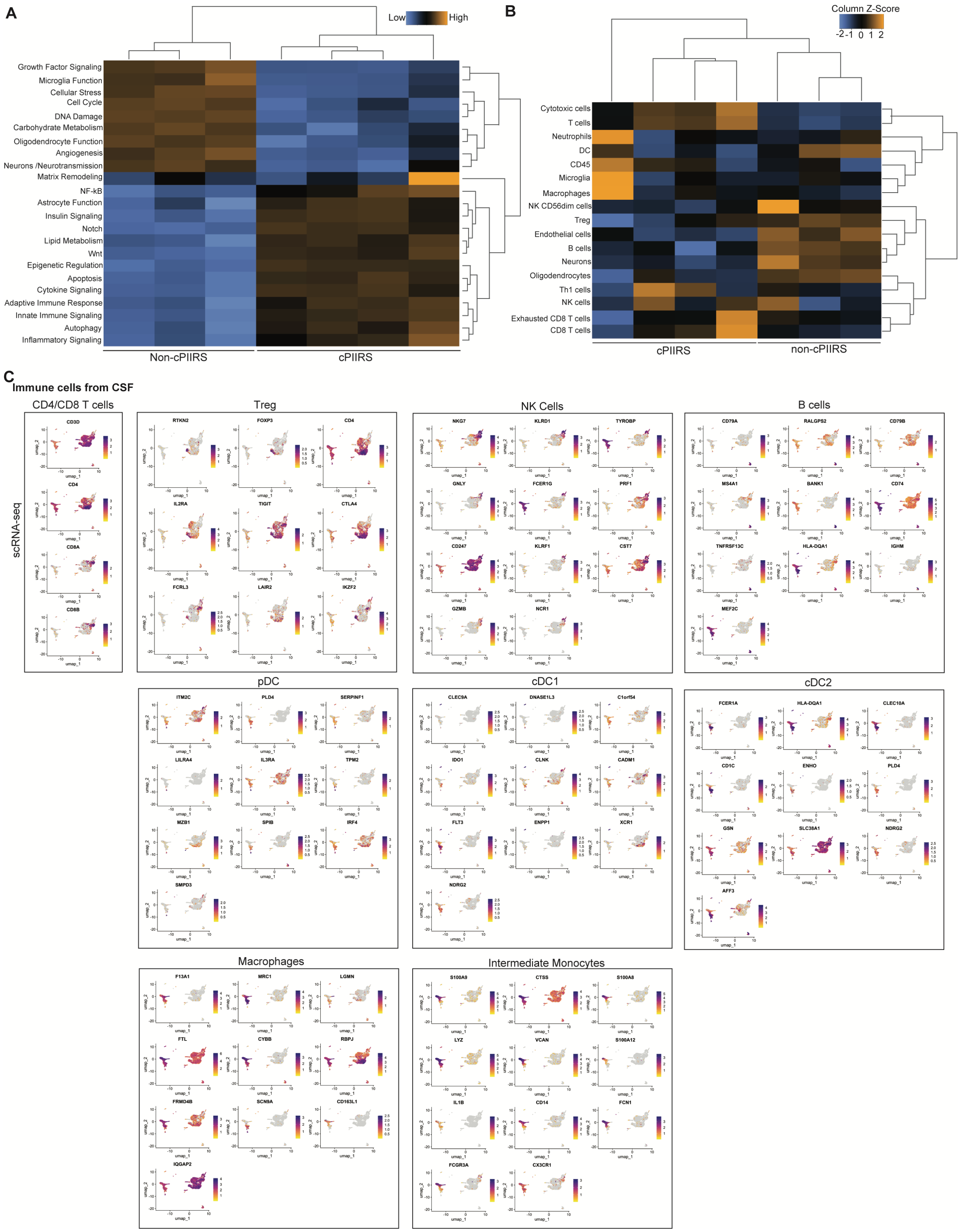

Fig S1. Transcriptional pathway analysis identifies the JAK/STAT inflammatory pathway as predominant in cPIIRS patient CSF. (A-B) Gene transcripts of cPIIRS patient CSF (at time of diagnosis) and non-cPIIRS donor CSF were analyzed using a NanoString multiplex neuroinflammation panel. **(A)** Heatmap of neuroinflammation pathway scores for cPIIRS patients compared to non-cPIIRS patient samples NanoString analysis. **(B)** Predicted CSF cell-type abundance was imputed from CSF mRNA obtained as in Fig. 1 using the NanoString Neuroinflammation Panel followed by gene expression and cell type abundance profiling. (n=4 cPIIRS patient (at time of diagnosis) and 3 non-cPIIRS donors CSF). (**C**) scRNA-seq analysis of CSF from cPIIRS patient at the time of cPIIRS diagnosis and prior to treatment. Selected genes corresponding to major cell lineages in UMAP embeddings of cells from cPIIRS patient CSF.

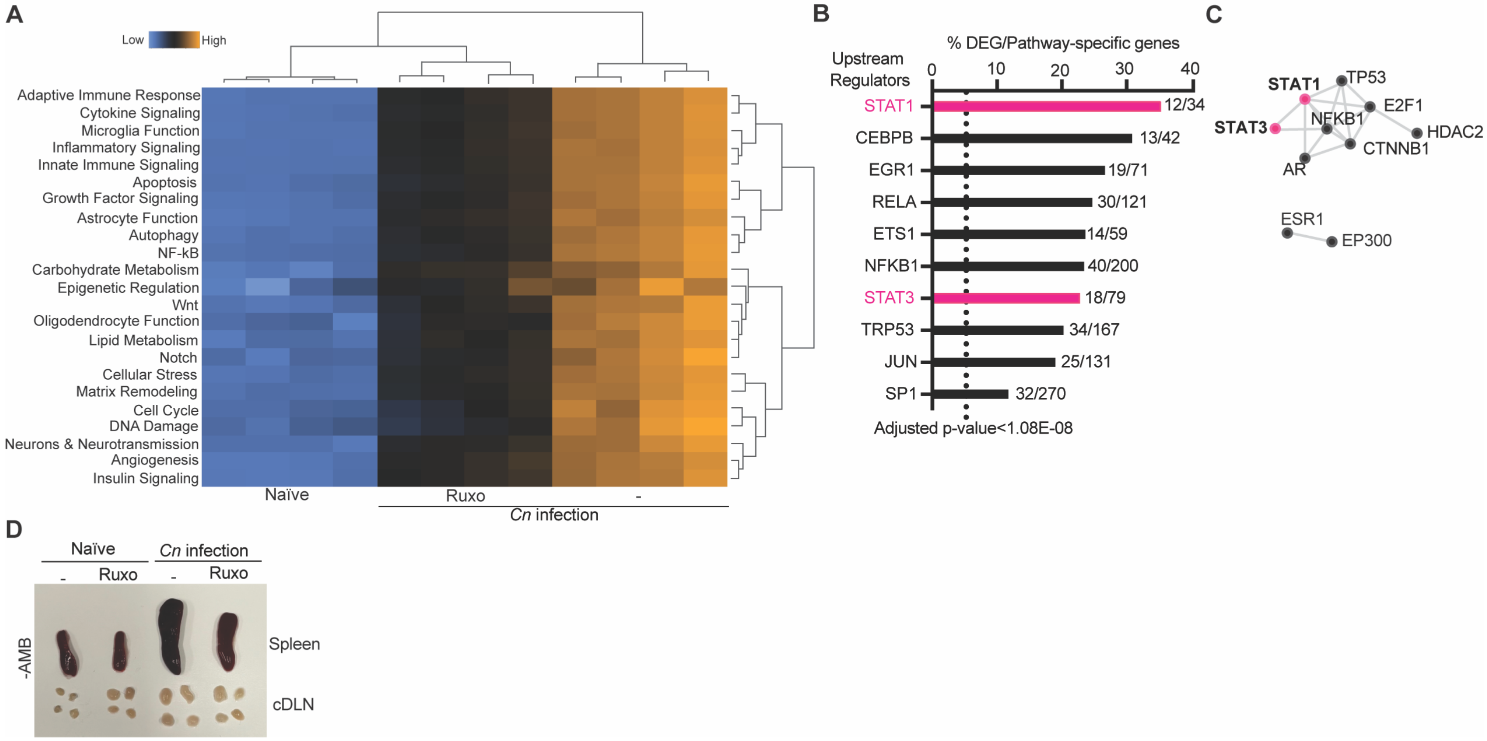

Figure S2. Transcriptional pathway analysis identifies the JAK/STAT inflammatory pathway as predominant in a cPIIRS mouse model with suppression by the JAK/STAT inhibitor ruxolitinib. (A) Heatmap of neuroinflammation pathway scores for naïve, *Cn* infected, and ruxolitinib-treated/*Cn* infected mouse brains by NanoString analysis. (**B-C)** Pathway analysis using differentially expressed genes between *Cn* infected and Naive mouse brain homogenates was performed using the Enrichr TRRUST Transcription Factors 2019 Database (B) and using the Enrichr Transcription Factor PPI to construct networks (C). Displayed are the top 10 regulators identified by each analysis tool based on adjusted -log10 p-value and graphed according to % of pathway-specific genes from mouse datasets identified in the DEGs. The fraction next to each bar shows the number of DEGs identified out of total number of genes regulated by each pathway according to the database. **(D)** Representative mouse spleen and cervical draining lymph node sizes at 21 dpi (infection model as in Fig. 2A).

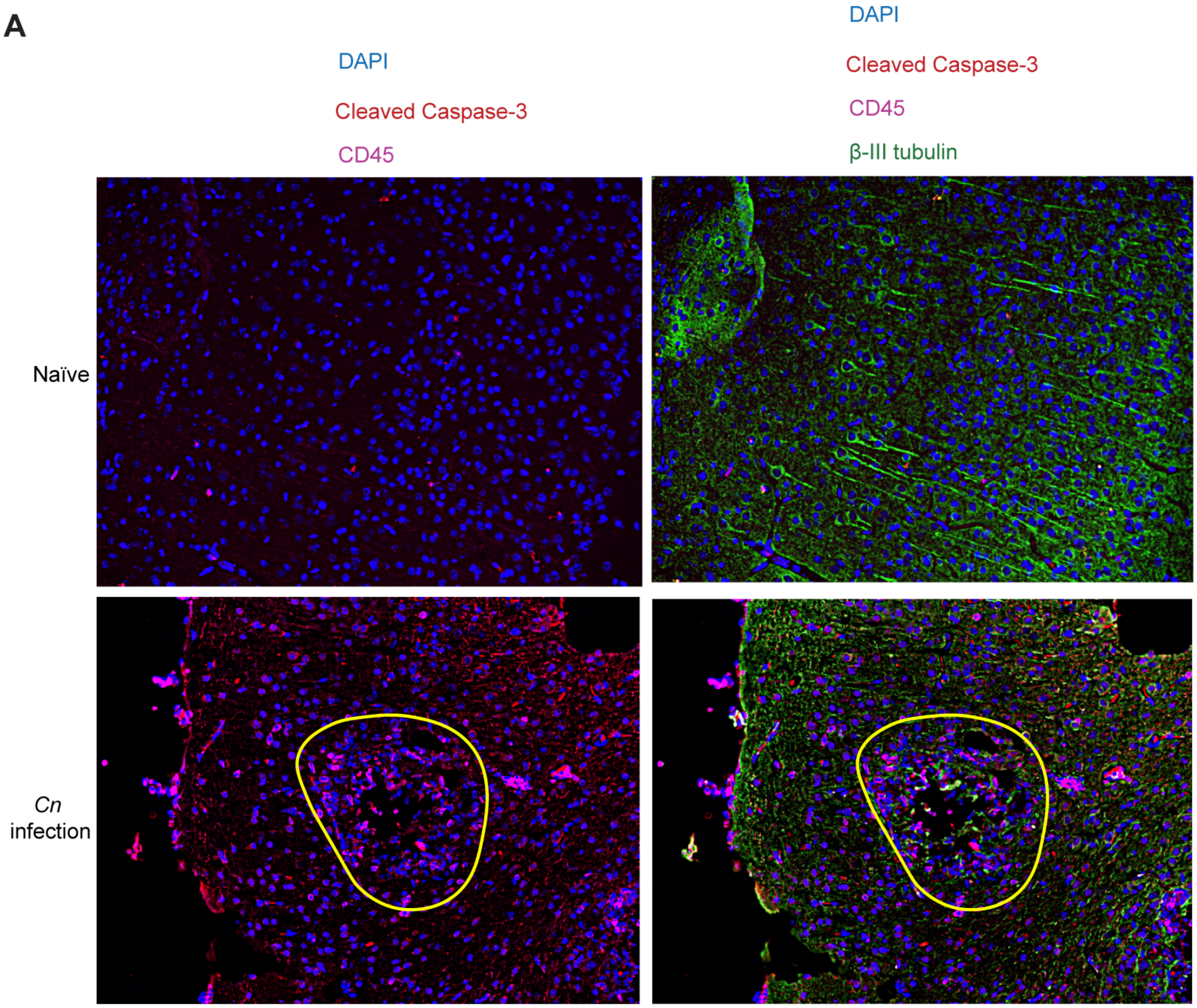

Figure S3. JAK/STAT-mediated inflammatory neurotoxicity is mitigated by ruxolitinib in a mouse model of cPIIRS. **(A)** IFM of brain sections stained with beta III tubulin (green), cleaved caspase-3 (red), CD45 (pink), and DAPI (blue). Note all ROIs (CD45 measurements) are from the cryptococcal lesion perimeter indicated by yellow circle. All cleaved caspase-3 measurements were taken from outside the perimeter. Areas that are 500 square microns around the cryptococcal lesions were analyzed. All the images were captured under the same settings.

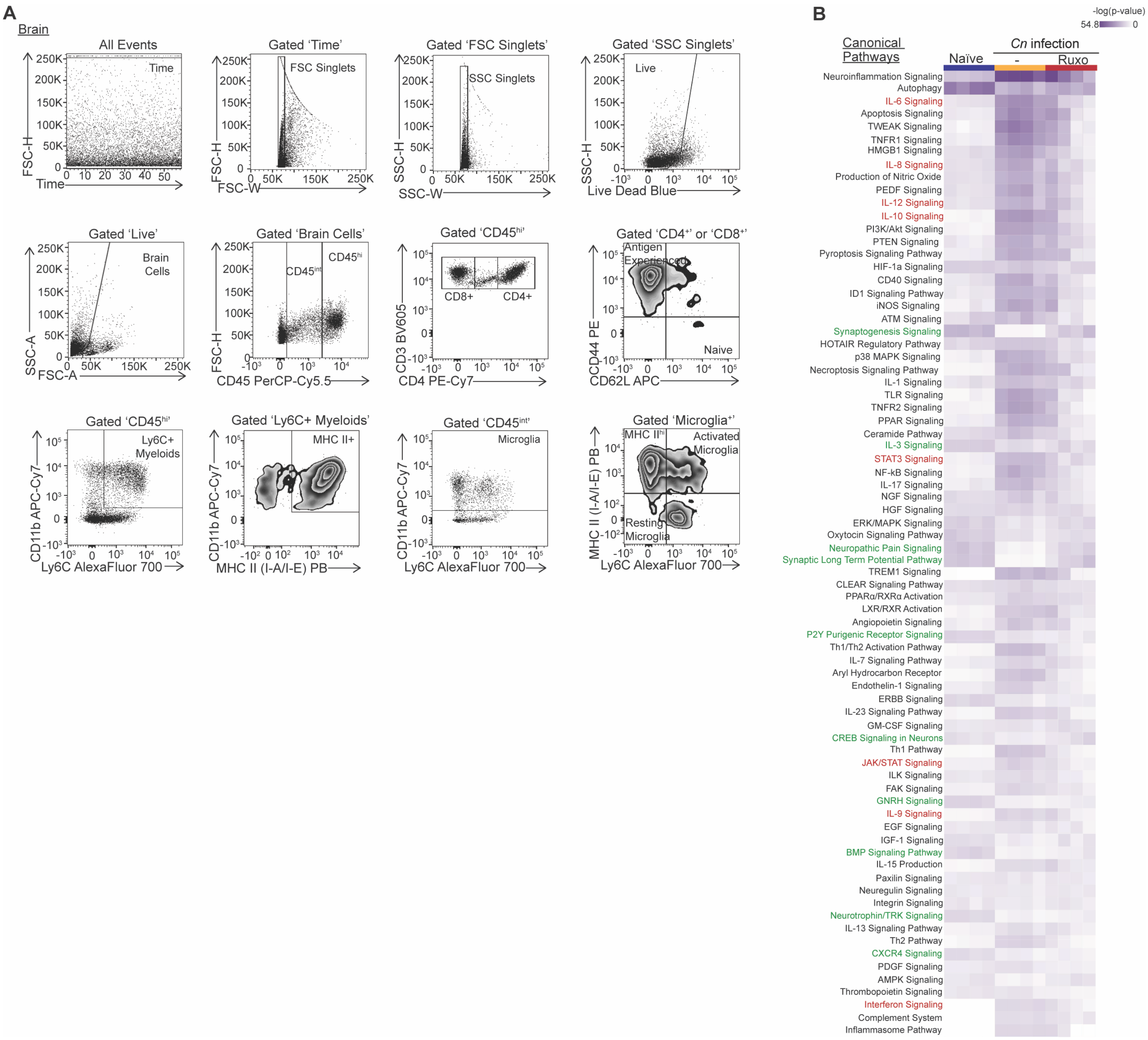

Figure S4. cPIIRS mouse brain flow cytometry gating strategy and IPA pathway analysis of NanoString data. (A) Gating strategy used to identify indicated brain immune populations as determined by flow cytometry. **(B)** Top 76 pathways identified using Qiagen IPA Pathway Analysis of DEGs between untreated/infected mice and other groups. Pathways labeled in green indicate pathways elevated in health and labeled in red indicate pathways related to JAK/STAT Signaling.

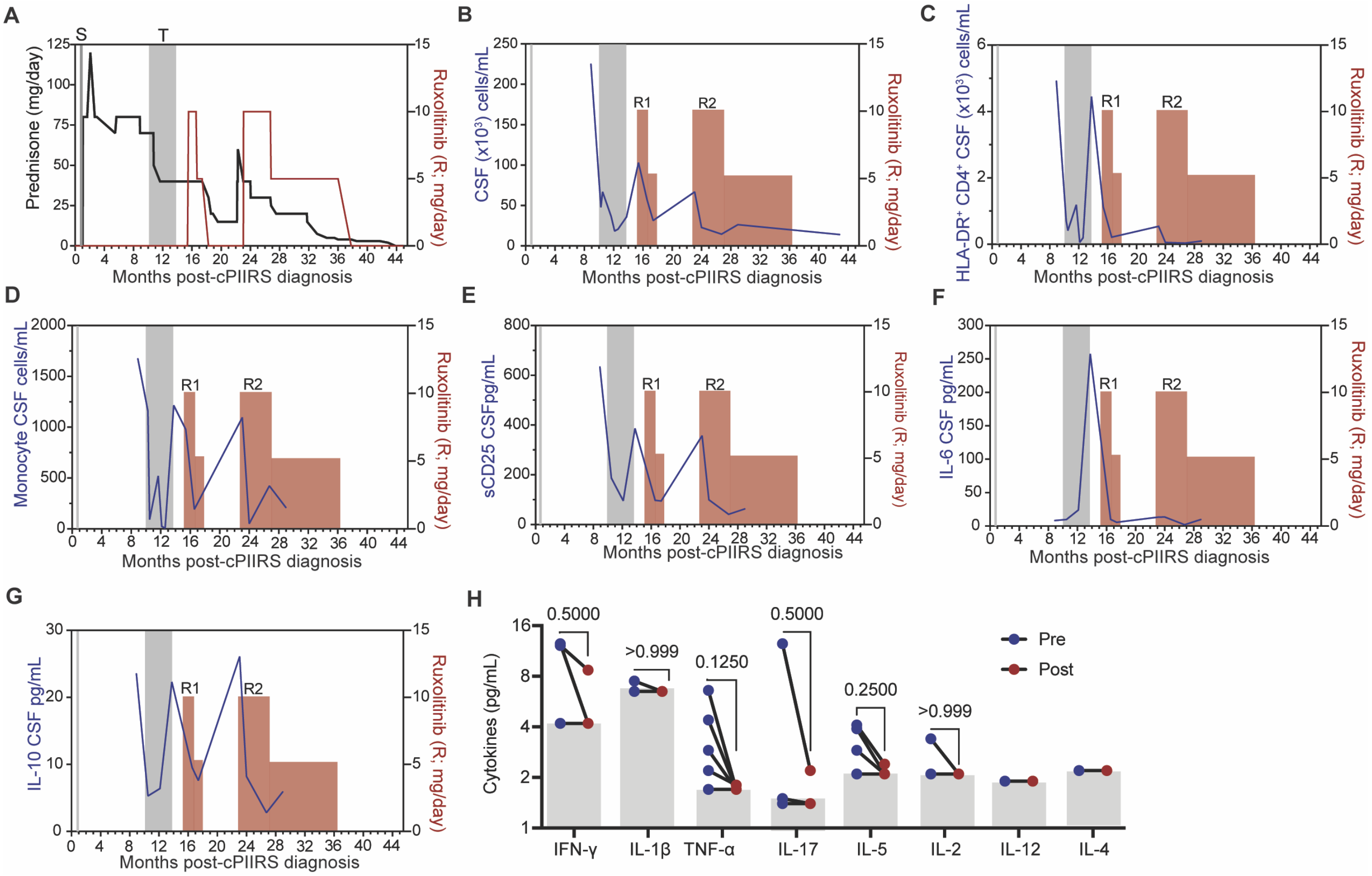

Figure S5. Representative patient treatment timeline (patient 1), longitudinal cerebrospinal fluid findings, and cytokine levels in patient CSF. (A) Following cPIIRS diagnosis, patient was treated with pulse solumedrol (S-vertical gray bar) followed by prednisone taper. Due to a rebound in headaches and mental status changes, the patient required an oral pulse of up to 120 mg of prednisone and a prolonged course of high doses of prednisone. The patient received intravenous infusions of tocilizumab (T-vertical gray bar) while maintained on prednisone until he experienced a rebound in these parameters after a delay in tocilizumab due to LFT abnormalities and tocilizumab was discontinued. Two courses of ruxolitinib (R#1 and R#2-red vertical bar) were initiated with reductions in CSF cell numbers (**B**), HLA-DR^{+} CD4 cells (**C**), monocytes (**D**), sCD25 (also known as soluble IL-2R) (**E**), IL-6 (**F**), and IL-10 (**G**) following each course of ruxolitinib which enabled a successful prednisone taper after the second course. **(H)** Concentrations of indicated soluble cytokines were measured from the CSF by a commercial assay as described in Methods**.** Data represents n=6 patients with 7 treatment courses, CSF cytokines analyzed using the Wilcoxon-matched pairs signed rank test, p-values indicated above each comparison.

Supplementary Table 1. Demographics and research evaluation information of the cPIIRS patients included in the present study. X signifies the contributions of each patient to this study.

Patient No.	Age at diagnosis	Race	Sex	CSF scRNA-seq (Fig. 1)	CSF NanoString (Fig. 1)	CSF Inflammation/ Neurological Findings (Fig. 5)
1	47-year-old	Caucasian	Male			X
2	58-year-old	African American	Male			X
3	60-year-old	Caucasian	Male		X	X
4	25-year-old	Caucasian	Male			X
5	43-year-old	Hispanic	Male			X
6	56-year-old	Caucasian	Female			X
7	75-year-old	Hawaiian/Pac. Islander	Female	X		
8	51-year-old	African American	Male		X	
9	72-year-old	Caucasian	Male		X	
10	45-year-old	Caucasian	Female		X	

Supplementary Table 2. Top 20 differentially expressed genes identified by NanoString analyses in CSF from cPIIRS patients compared to non-cPIIRS controls, with Log2 fold-change > 0.58 and p<0.05.

Gene name	Biological Process	Log2FC	Lower	Upper	p value
CD47	Autophagy, Matrix Remodeling	5.68	5.6	5.76	3.10E-10
TOPBP1	DNA Damage	5.6	5.49	5.71	1.89E-09
MAVS	Inflammatory Signaling, Innate Immune Response	4.67	4.56	4.77	3.69E-09
XRCC6	DNA Damage, Innate Immune Response	6.6	6.44	6.76	6.14E-09
CASP2	Apoptosis, Growth Factor Signaling, Innate Immune Response	6.28	6.07	6.48	2.62E-08
EZH1	Epigenetic Regulation	4.35	4.16	4.55	1.17E-07
RAD50	Cell Cycle, Cellular Stress, DNA Damage	5.64	5.37	5.9	1.49E-07
KAT2A	Epigenetic Regulation, Notch	5.62	5.31	5.93	3.46E-07
MDC1	Cell Cycle, DNA Damage	5.04	4.7	5.38	9.33E-07
APEX1	Cell Cycle, DNA Damage	4.69	4.35	5.02	1.17E-06
ICAM2	Adaptive Immune Response, Innate Immune Response, Matrix Remodeling	4.93	4.57	5.3	1.49E-06
UNG	DNA Damage	4.39	3.99	4.78	3.84E-06
DNA2	Cell Cycle, DNA Damage	2.09	1.86	2.31	9.41E-06
TMEM206	Microglia Function	3.39	3.02	3.75	9.53E-06
PRKCE	Autophagy, Growth Factor Signaling	4.1	3.58	4.63	2.13E-05
CLSTN1	Microglia Function	3.29	2.86	3.72	2.39E-05
NOD1	Autophagy, Cytokine Signaling, Innate Immune Response	3.56	2.99	4.13	6.66E-05
SIRT1	Carbohydrate Metabolism, Cellular Stress, Epigenetic Regulation	3.42	2.86	3.98	7.28E-05
FEN1	Cell Cycle, DNA Damage	3.46	2.89	4.03	7.29E-05
TIMELESS	Cell Cycle, DNA Damage	3.6	3.01	4.2	7.54E-05

Supplementary Table 3. Top 20 differentially expressed genes identified by NanoString analyses in brain homogenates from mice infected and treated with ruxolitinib at 21 dpi compared to infected untreated controls, with Log2 fold-change > 0.58 and p<0.05.

Gene name	Biological Process	Log2FC	Lower	Upper	p value
Cd3g	Adaptive Immune Response	-3.56	-3.91	-3.21	9.05E-09
Nkg7	Cytotoxic Lymphocytes	-3.41	-3.78	-3.05	1.79E-08
Lilrb4a	Adaptive Immune Response, Inflammatory Signaling	-2.41	-2.67	-2.14	2.62E-08
Ptprc	Adaptive Immune Response, Matrix Remodeling	-2.27	-2.54	-2	4.91E-08
Gadd45g	Cell Cycle, DNA Damage, Growth Factor Signaling	-1.04	-1.17	-0.916	5.87E-08
Hmox1	Cellular Stress	-2.57	-2.88	-2.25	5.99E-08
Pros1	Autophagy	-0.98	-1.11	-0.852	1.10E-07
Pla2g4a	Autophagy, Cellular Stress, Growth Factor Signaling, Lipid Metabolism, Oligodendrocyte Function	-1.43	-1.63	-1.24	1.57E-07
Cd86	Adaptive Immune Response, Growth Factor Signaling, Matrix Remodeling, Microglia Function	-2.07	-2.35	-1.78	1.82E-07
Srgn	Astrocyte Function, Inflammatory Signaling	-2.13	-2.43	-1.83	1.99E-07
Atp6v0e	Growth Factor Signaling, Insulin Signaling, Microglia Function	-0.883	-1.01	-0.758	2.13E-07
C5ar1	Inflammatory Signaling, Neurons and Neurotransmission	-1.92	-2.2	-1.65	2.48E-07
C3	Adaptive Immune Response, Autophagy, Inflammatory Signaling, Innate Immune Response, Microglia Function	-2.68	-3.07	-2.3	2.59E-07
Dock2	Autophagy	-1.53	-1.75	-1.3	2.88E-07
Fcgr2b	Adaptive Immune Response, Autophagy	-1.81	-2.08	-1.55	3.18E-07
Mapk10	Cytokine Signaling, Inflammatory Signaling, NF-kB	0.24	0.204	0.276	3.60E-07
Tnfrsf12a	Adaptive and Innate Immune Response, Apoptosis, Autophagy, Cellular Stress, Growth Factor Signaling, Wnt	-0.891	-1.02	-0.758	3.60E-07
Lamp2	Autophagy	-0.891	-1.03	-0.757	3.88E-07
Cp	Astrocyte Function	-1.27	-1.46	-1.08	3.96E-07
Casp4	Apoptosis, Innate Immune Response	-2.36	-2.72	-2	4.40E-07

Supplementary Table 4. Top 20 differentially expressed genes identified by NanoString analyses in brain homogenates from Naïve mice compared to infected untreated controls at 21 dpi, with Log2 fold-change > 0.58 and p<0.05.

Gene name	Biological Process	Log2FC	Lower	Upper	p value
Cd74	Adaptive Immune Response, Inflammatory Signaling	-8.2	-8.54	-7.86	4.80E-12
Lilrb4a	Adaptive Immune Response, Inflammatory Signaling	-6.8	-7.13	-6.47	1.65E-11
C3	Adaptive and Innate Immune Response, Autophagy, Inflammatory Signaling, Microglia Function	-7.4	-7.82	-6.99	6.41E-11
Bcl2a1a	Apoptosis, NF-kB	-6.02	-6.37	-5.67	8.57E-11
C1qb	Innate Immune Response	-3.73	-3.94	-3.51	8.62E-11
C1qa	Innate Immune Response	-3.92	-4.15	-3.69	9.97E-11
Fcgr2b	Adaptive Immune Response, Autophagy	-4.71	-4.99	-4.43	1.03E-10
C1qc	Innate Immune Response	-3.78	-4.01	-3.56	1.14E-10
Ptprc	Adaptive Immune Response, Matrix Remodeling	-5.05	-5.36	-4.75	1.33E-10
Ctss	Adaptive Immune Response, Innate Immune Response, Matrix Remodeling, Microglia Function	-4.29	-4.56	-4.03	1.39E-10
Gbp2	Astrocyte Function, Inflammatory Signaling	-7.82	-8.31	-7.33	1.59E-10
Mpeg1	Inflammatory Signaling	-4.79	-5.09	-4.49	1.70E-10
Tyrobp	Adaptive Immune Response, Innate Immune Response	-3.26	-3.47	-3.05	1.88E-10
Dock2	Autophagy	-3.95	-4.2	-3.7	2.11E-10
C4a	Astrocyte Function	-4.38	-4.66	-4.09	2.70E-10
Ccl5	Cellular Stress, Cytokine Signaling, Inflammatory Signaling, Innate Immune Response, Microglia Function	-10	-10.7	-9.34	3.62E-10
Il2rg	Adaptive Immune Response, Angiogenesis, Cytokine Signaling, Growth Factor Signaling, Inflammatory Signaling, Insulin Signaling	-6.36	-6.8	-5.92	4.13E-10
Psmb8	Adaptive Immune Response, Angiogenesis, Apoptosis, Astrocyte Function, Cell Cycle, Cytokine Signaling, Growth Factor Signaling, Inflammatory Signaling, Insulin Signaling, Microglia Function, NF-kB, Wnt	-6.28	-6.72	-5.84	4.68E-10
Pros1	Autophagy	-1.8	-1.93	-1.67	5.14E-10
Fcer1g	Adaptive Immune Response, Autophagy, Inflammatory Signaling, Innate Immune Response	-4.12	-4.42	-3.83	6.11E-10

Supplementary Table 5. Demographics and characteristics of patients with post-infectious inflammatory response syndrome treated with ruxolitinib.

Patient No.	Age at diagnosis<br>Race<br>Sex<br>*Cryptococcus*	Baseline CD4 counts (cells/uL)	Presenting symptoms	MRI brain findings at cPIIRS diagnosis	Co-morbidities	Corticosteroid Treatment Duration (months)	Ruxolitinib Treatment Duration (months)
1	47-year-old<br>Caucasian<br>Male<br>*C. gattii*	414	Headaches, hearing loss	Hyperintense oval mass in the right superior frontal lobe with eccentric enhancement	Anxiety, Depression, Hypothyroidism, Alcohol use, Hypertension, Osteopenia	33	#1: 3<br>#2: 14
2	58-year-old<br>African American<br>Male<br>*C. neoformans*	309	Altered mental status	Leptomeningeal enhancement, communicating hydrocephalus, arachnoiditis	Type II Diabetes Mellitus, Ischemic Heart Disease, Chronic Kidney Disease	19	2
3	60-year-old<br>Caucasian<br>Male<br>Unknown	561	Headaches, hearing loss, gait instability	Focal leptomeningeal enhancement within the superior frontal sulcus, enhancement within the internal auditory canals bilaterally	None	15	3
4	25-year-old<br>Caucasian<br>Male<br>*C. grubii* VNI	1103	Headaches, hearing loss, blurred vision	Multiple peri-ventricular and subcortical white matter hyperintense lesions, enhancement in the internal auditory canals	Opioid and Marijuana use disorder	2	2
5	43-year-old<br>Hispanic<br>Male<br>Unknown	741	Headaches, Double vision, gait instability	Ventriculomegaly, periventricular edema, communicating hydrocephalus, ependymitis, ventriculitis	Positive serology for South American Trypanosomiasis	None	5
6	56-year-old<br>Caucasian<br>Female<br>Unknown	503	Headaches, Hearing loss, dizziness, altered mental status	Diffuse meningeal enhancement, ventriculomegaly, enhancement along the internal auditory canals	Diverticulitis, Fibromyalgia, Deep Vein Thrombosis, bilateral optic neuropathy	18 (discontinued prior to Ruxo)	2

Supplementary Table 6. Summary of patient complications due to cPIIRS and prolonged corticosteroid therapy and other adjunctive therapies tested.

Description	Time of event		% of patients impacted (Patient #)
	cPIIRS Diagnosis	During corticosteroid therapy	
**Complications:**			
VP shunt inserted for elevated intracranial pressure	Yes		1/6 (Patient 1)
> 6 months corticosteroid therapy		Yes	4/6
Developed worsening cataracts and osteopenia		Yes	3/6
Developed steroid-induced psychosis, hallucinations, and worsening hyperglycemia		Yes	1/6
**Other Adjunctive Therapy Tested:**			
IL-6 receptor alpha (IL-6Rα) antagonist tocilizumab with 4 doses (4-8 mg/kg) given over 4 months (*79*), but discontinued due to liver enzyme abnormalities		Yes	1/6 (Patient 1)

Supplementary Table 7. Laboratory parameters of patients before (pre) and one month (post) after starting Ruxolitinib treatment. Red indicates abnormal values.

Parameter	Patient 1				Patient 2		Patient 3		Patient 4		Patient 5		Patient 6		Normal Range
	Pre#1	Post#1	Pre#2	Post#2	Pre	Post	Pre	Post	Pre	Post	Pre	Post	Pre	Post	
Red Blood Cell, m/mcL	4.47	4.48	4.81	4.38	3.44	3.1	3.13	3.19	4.29	4.47	3.47	3.19	4.08	4.19	4.63 – 6.08
**Hemoglobin, g/dL**	**14.3**	**14.4**	**14.3**	**13.4**	**9.7**	**8.7**	**10**	**11.3**	**12**	**12.3**	**10.2**	**9.5**	**12.2**	**12.4**	**13.7 – 17.5**
Mean Cell Volume, fL	95.1	92.2	90.4	92	87.5	86.1	94.9	97.8	86	84.1	82.4	86.2	94.1	91.4	79 – 92.2
Mean Cell Hemoglobin, pg	32.0	32.1	29.7	30.6	28.8	28.1	31.9	35.4	28	27.5	29.4	29.8	29.9	29.6	25.7 – 32.2
**Platelet, K/mcL**	**200**	**303**	**228**	**278**	**338**	**340**	**146**	**191**	**362**	**521**	**182**	**220**	**332**	**479**	**161 - 347**
White Blood Cell, K/mcL	6.73	9.14	6.09	8.74	13.25	7.39	10.72	9.93	13.58	10.78	4.39	3.65	4.85	3.85	4.23 – 9.07
Absolute Neutrophil count, K/mcL	4.53	5.76	3.59	6.48	9.63	6.11	5.71	7.76	9.3	8.24	1.84	1.55	2.49	1.54	1.78 – 5.38
Absolute Lymphocyte Count,K/mcL	1.36	2.86	1.87	1.26	0.99	0.32	3.57	1.53	3.17	1.83	2.13	1.8	1.85	1.98	1.32 – 3.57
Monocyte count, K/mcL	0.66	0.35	0.46	0.85	2.22	0.75	0.6	0.0	0.7	0.65	0.31	0.21	0.35	0.26	0.3 – 0.82
Eosinophils, K/mcL	0.05	0.03	0.07	0.03	0.05	0.00	0.1	0.00	0.17	0.03	0.04	0.06	0.10	0.05	0.04 – 0.54
Basophils, K/mcL	0.05	0.03	0.04	0.04	0.03	0.01	0.00	0.00	0.03	0.01	0.05	0.01	0.05	0.01	0.01 – 0.08
Immature granulocytes, K/mcL	0.08	0.11	0.06	0.08	0.33	0.20	-	-	0.21	0.02	0.02	0.02	0.01	0.01	0.00 – 0.03
Alkaline phosphatase, U/L	72	56	50	71	168	97	42	36	65	73	100	93	108	115	40 - 150
Aspartate Aminotransferase, U/L	103	212	29	28	33	20	12	15	14	16	27	23	16	20	5 - 34
Alanine Aminotransferase, U/L	152	239	45	33	77	56	26	30	28	27	99	32	12	16	<55
Total Bilirubin, mg/dL	0.8	0.6	0.3	0.3	0.3	0.2	0.3	0.3	0.2	0.2	0.5	0.7	0.2	0.4	0.2 – 1.2
Direct bilirubin, mg/dL	0.3	0.3	0.1	0.1	0.2	0.1	0.1	0.1	<0.1	0.1	0.1	0.2	0.1	0.1	<0.5
Albumin, g/dL	4.5	4.6	4.3	4.3	3.2	3.3	4.0	4.3	3.9	4.2	4.0	4.5	4.2	4.6	3.5 – 5.2
Creatinine, mg/dL	0.68	0.92	0.83	0.73	1.47	1.92	0.99	1.13	0.85	0.84	1.17	1.13	0.89	0.97	0.73 – 1.18
Urea Nitrogen, mg/dL	16	25	17	12	28	49	23	23	20	15	23	35	13	16	9 – 21
Potassium, mmol/L	4.2	3.7	4.1	4.1	4.8	4.9	4.1	4.5	3.6	4.1	3.6	3.7	4.2	4.1	3.5 – 5.1
Sodium, mmol/L	137	137	143	140	133	137	136	137	139	138	143	139	138	135	136 - 145
Calcium, mg/dL	9.1	8.6	9.1	9.4	8.9	8.6	9.6	9.5	9.0	9.7	8.8	9.3	9.3	9.2	8.4 – 10.2
Phosphorus, mg/dL	2.9	2.6	2.7	3.4	2.8	2.4	3.0	3.0	2.8	2.6	4.8	3.9	3.1	3.0	2.3 – 4.7
Magnesium, mg/dL	1.8	2.0	2.0	1.9	1.8	2.0	2.0	2.0	2.0	2.1	1.0	1.8	1.8	1.9	1.6 – 2.6
Random blood sugar, mg/dL	99	96	95	117	167	297	103	246	137	122	101	111	113	84	70 - 99

REFERENCES AND NOTES

1. L.-A. Pirofski, A. Casadevall, Pathogenesis of COVID-19 from the perspective of the damage-response framework. *mBio* **11**, e01175-20 (2020).

2. A. A. Panackal, K. C. Williamson, D. van de Beek, D. R. Boulware, P. R. Williamson, Fighting the monster: Applying the host damage framework to human central nervous system infections *mBio* **7**, e01906–e01915 (2016).

3. V. M. S. Brienze, J. C. Andre, E. Liso, I. V.-St Louis, Cryptococcal immune reconstitution inflammatory syndrome: From blood and cerebrospinal fluid biomarkers to treatment approaches. *Life (Basel)* **11**, 95 (2021).

4. A. A. Panackal, S. C. Wuest, Y.-C. Lin, T. Wu, N. Zhang, P. Kosa, M. Komori, A. Blake, S. K. Browne, L. B. Rosen, F. Hagen, J. Meis, S. M. Levitz, M. Quezado, D. Hammoud, J. E. Bennett, B. Bielekova, P. R. Williamson, Paradoxical immune responses in non-HIV cryptococcal meningitis. *PLOS Pathog.* **11**, e1004884 (2015).

5. Meya D. B. Williamson P. R. Cryptococcal disease in diverse hosts *N. Engl. J. Med.* **390** 1597 1610 2024

6. K. Ssebambulidde, S. H. Anjum, J. C. Hargarten, P. Chittiboina, S. Shoham, S. Seyedmousavi, K. A. Marr, D. A. Hammoud, B. J. Billioux, P. R. Williamson, Treatment recommendations for non-HIV associated cryptococcal meningoencephalitis including management of post-infectious inflammatory response syndrome. *Front. Neurol.* **13**, 994396 (2022).

7. A. Balasko, Y. Keynan, Shedding light on IRIS: From pathophysiology to treatment of cryptococcal meningitis and immune reconstitution inflammatory syndrome in HIV-infected individuals. *HIV Med.* **20** 1–10 (2019).

8. S. Anjum, O. Dean, P. Kosa, M. T. Magone, K. A. King, E. Fitzgibbon, H. J. Kim, C. Zalewski, E. Murphy, B. J. Billioux, J. Chisholm, C. C. Brewer, C. Krieger, W. Elsegeiny, T. L. Scott, J. Wang, S. Hunsberger, J. E. Bennett, A. Nath, K. A. Marr, B. Bielekova, D. Wendler, D. A. Hammoud, P. Williamson, Outcomes in previously healthy cryptococcal meningoencephalitis

patients treated with pulse taper corticosteroids for post-infectious inflammatory syndrome. *Clin. Infect. Dis.* **73**, e2789–e2798 (2021).

9. G. Meintjes, C. Stek, L. Blumenthal, F. Thienemann, C. Schutz, J. Buyze, R. Ravinetto, H. van Loen, A. Nair, A. Jackson, R. Colebunders, G. Maartens, R. J. Wilkinson, L. Lynen, PredART Trial Team, Prednisone for the prevention of paradoxical tuberculosis-associated IRIS. *N. Engl. J. Med.* **379**, 1915–1925 (2018).

10. N. Duru, M. C. van der Goes, J. W. Jacobs, T. Andrews, M. Boers, F. Buttgereit, N. Caeyers, M. Cutolo, S. Halliday, J. A. Da Silva, J. R. Kirwan, D. Ray, J. Rovensky, G. Severijns, R. Westhovens, J. W. Bijlsma, EULAR evidence-based and consensus-based recommendations on the management of medium to high-dose glucocorticoid therapy in rheumatic diseases. *Ann. Rheum. Dis.* **72**, 1905–1913 (2013).

11. O. Butovsky, M. P. Jedrychowski, R. Cialic, S. Krasemann, G. Murugaiyan, Z. Fanek, D. J. Greco, P. M. Wu, C. E. Doykan, O. Kiner, R. J. Lawson, M. P. Frosch, N. Pochet, R. E. Fatimy, A. M. Krichevsky, S. P. Gygi, H. Lassmann, J. Berry, M. E. Cudkowicz, H. L. Weiner, Targeting miR-155 restores abnormal microglia and attenuates disease in SOD1 mice. *Ann. Neurol.* **77**, 75–99 (2015).

12. Y. Wang, M. Cella, K. Mallinson, J. D. Ulrich, K. L. Young, M. L. Robinette, S. Gilfillan, G. M. Krishnan, S. Sudhakar, B. H. Zinselmeyer, D. M. Holtzman, J. R. Cirrito, M. Colonna, TREM2 lipid sensing sustains the microglial response in an Alzheimer's disease model. *Cell* **160**, 1061–1071 (2015).

13. K. Srinivasan, B. A. Friedman, J. L. Larson, B. E. Lauffer, L. D. Goldstein, L. L. Appling, J. Borneo, C. Poon, T. Ho, F. Cai, P. Steiner, Marcel P van der Brug, Z. Modrusan, J. S. Kaminker, D. V. Hansen, Untangling the brain's neuroinflammatory and neurodegenerative transcriptional responses. *Nat. Commun.* **7**, 11295 (2016).

14. A. Liberzon, C. Birger, H. Thorvaldsdottir, M. Ghandi, J. P. Mesirov, P. Tamayo, The Molecular Signatures Database (MSigDB) hallmark gene set collection. *Cell Syst.* **1**, 417–425 (2015).

15. R. L. Philips, Y. Wang, H. Cheon, Y. Kanno, M. Gadina, V. Sartorelli, C. M. Horvath, J. E. Darnell Jr., G. R. Stark, J. J. O'Shea, The JAK-STAT pathway at 30: Much learned, much more to do. *Cell* **185**, 3857–3876 2022

16. L. M. Neal, E. Xing, J. Xu, J. L. Kolbe, J. J. Osterholzer, B. M. Segal, P. R. Williamson, M. A. Olszewski, $CD4^+$ T cells orchestrate lethal immune pathology despite fungal clearance during Cryptococcus neoformans meningoencephalitis. *mBio* **8**, e01415–e01417 (2017).

17. W. C. Uicker, H. A. Doyle, J. P. McCracken, M. Langlois, K. L. Buchanan, Cytokine and chemokine expression in the central nervous system associated with protective cell-mediated immunity against Cryptococcus neoformans. *Med. Mycol.* **43**, 27–38 (2005).

18. W. C. Uicker, J. P. McCracken, K. L. Buchanan, Role of $CD4^+$ T cells in a protective immune response against *Cryptococcus neoformans* in the central nervous system. *Med. Mycol.* **44**, 1–11 (2006).

19. K. L. Buchanan, H. A. Doyle, Requirement for $CD4^+$ T lymphocytes in host resistance against *Cryptococcus neoformans* in the central nervous system of immunized mice. *Infect. Immun.* **68**, 456–462 (2000).

20. F. Chrétien, O. Lortholary, I. Kansau, S. Neuville, F. Gray, F. Dromer, Pathogenesis of cerebral *Cryptococcus neoformans* infection after fungemia. *J. Infect. Dis.* **186**, 522–530 (2002).

21. Q. Zhou, R. A. Gault, T. R. Kozel, W. J. Murphy, Protection from direct cerebral cryptococcus infection by interferon-γ-dependent activation of microglial cells. *J. Immunol.* **178**, 5753–5761 (2007).

22. A. Pool, L. Lowder, Y. Wu, K. Forrester, J. Rumbaugh, Neurovirulence of Cryptococcus neoformans determined by time course of capsule accumulation and total volume of capsule in the brain *J. Neurovirol.* **19**, 228–238 (2013).

23. P. Danaher, Y. Kim, B. Nelson, M. Griswold, Z. Yang, E. Piazza, J. M. Beechem, Advances in mixed cell deconvolution enable quantification of cell types in spatial transcriptomic data *Nat. Commun.* **13**, 385 (2022).

24. I. M. Chiu, E. T. Morimoto, H. Goodarzi, J. T. Liao, S. O'Keeffe, H. P. Phatnani, M. Muratet, M. C. Carroll, S. Levy, S. Tavazoie, R. M. Myers, T. Maniatis, A neurodegeneration-specific gene-expression signature of acutely isolated microglia from an amyotrophic lateral sclerosis mouse model. *Cell Rep.* **4**, 385–401 (2013).

25. E. Senkevitch, W. Li, J. A. Hixon, C. Andrews, S. D. Cramer, G. T. Pauly, T. Back, K. Czarra, S. K. Durum, Inhibiting Janus Kinase 1 and BCL-2 to treat T cell acute lymphoblastic leukemia with IL7-Rα mutations. *Oncotarget* **9**, 22605–22617 (2018).

26. V. Oikonomou, G. Smith, G. M. Constantine, M. M. Schmitt, E. M. N. Ferre, J. C. Alejo, D. Riley, D. Kumar, L. Dos Santos Dias, J. Pechacek, Y. Hadjiyannis, T. Webb, B. A. Seifert, R. Ghosh, M. Walkiewicz, D. Martin, M. Besnard, B. D. Snarr, S. Deljookorani, C. R. Lee, T. DiMaggio, P. Barber, L. B. Rosen, A. Cheng, A. Rastegar, A. A. de Jesus, J. Stoddard, H. S. Kuehn, T. J. Break, H. H. Kong, L. Castelo-Soccio, B. Colton, B. M. Warner, D. E. Kleiner, M. M. Quezado, J. L. Davis, K. P. Fennelly, K. N. Olivier, S. D. Rosenzweig, A. F. Suffredini, M. S. Anderson, M. Swidergall, C. Guillonneau, L. D. Notarangelo, R. Goldbach-Mansky, O. Neth, M. T. Monserrat-Garcia, J. Valverde-Fernandez, J. M. Lucena, A. L. Gomez-Gila, A. Garcia Rojas, M. R. J. Seppanen, J. Lohi, M. Hero, S. Laakso, P. Klemetti, V. Lundberg, O. Ekwall, P. Olbrich, K. K. Winer, B. Afzali, N. M. Moutsopoulos, S. M. Holland, T. Heller, S. Pittaluga, M. S. Lionakis, The role of interferon-γ in autoimmune polyendocrine syndrome type 1. *N. Engl. J. Med.* **390**, 1873–1884 (2024).

27. J. Xu, A. Ganguly, J. Zhao, M. Ivey, R. Lopez, J. J. Osterholzer, C. S. Cho, M. A. Olszewski, CCR2 signaling promotes brain infiltration of inflammatory monocytes and contributes to neuropathology during cryptococcal meningoencephalitis *mBio* **12**, e0107621 (2021).

28. J. Xu, L. M. Neal, A. Ganguly, J. L. Kolbe, J. C. Hargarten, W. Elsegeiny, C. Hollingsworth, X. He, M. Ivey, R. Lopez, J. Zhao, B. Segal, P. R. Williamson, M. A. Olszewski, Chemokine receptor CXCR3 is required for lethal brain pathology but not pathogen clearance during cryptococcal meningoencephalitis. *Sci. Adv.* **6,** eaba2502 (2020).

29. K. Sau, S. S. Mambula, E. Latz, P. Henneke, D. T. Golenbock, S. M. Levitz, The antifungal drug amphotericin B promotes inflammatory cytokine release by a Toll-like receptor- and CD14-dependent mechanism. *J. Biol. Chem.* **278,** 37561–37568 (2003).

30. A. C. Mesa-Arango, L. Scorzoni, O. Zaragoza, It only takes one to do many jobs: Amphotericin B as antifungal and immunomodulatory drug. *Front. Microbiol.* **3**, 286 (2012).

31. R. Lu, C. Hollingsworth, J. Qiu, A. Wang, E. Hughes, X. Xin, K. M. Konrath, W. Elsegeiny, Y. D. Park, L. Atakulu, J. C. Craft, E. C. Tramont, R. Mannino, P. R. Williamson, Efficacy of oral encochleated amphotericin B in a mouse model of cryptococcal meningoencephalitis. *mBio* **10**, e00724-19 (2019).

32. G. Bindea, B. Mlecnik, M. Tosolini, A. Kirilovsky, M. Waldner, A. C. Obenauf, H. Angell, T. Fredriksen, L. Lafontaine, A. Berger, P. Bruneval, W. H. Fridman, C. Becker, F. Pagès, M. R. Speicher, Z. Trajanoski, J. Galon, Spatiotemporal dynamics of intratumoral immune cells reveal the immune landscape in human cancer. *Immunity* **39**, 782–795 (2013).

33. M. da Consolação Vieira Moreira, J. R. Cunha-Melo, Chagas disease infection reactivation after heart transplant. *Trop. Med. Infect. Dis.* **5**, 106 (2020).

34. S. J. Hurwitz, S. Tao, C. Gavegnano, Y. Jiang, R. L. Tressler, A. Tsibris, C. Del Rio, E. T. Overton, M. M. Lederman, A. Kantor, C. Moser, J. J. Kohler, J. Lennox, V. C. Marconi, C. W. Flexner, R. F. Schinazi, Pharmacokinetics of ruxolitinib in HIV suppressed individuals on antiretroviral agent therapy from the ACTG A5336 study. *J. Clin. Pharmacol.* **61**, 1555–1566 (2021).

35. L. Pizzamiglio, E. Focchi, F. Antonucci, ATM protein kinase: Old and new implications in neuronal pathways and brain circuitry. *Cells* **9**, 1969 (2020).

36. B. Bielecki, C. Mattern, A. M. Ghoumari, S. Javaid, K. Smietanka, C. Abi Ghanem, S. Mhaouty-Kodja, M. S. Ghandour, E. E. Baulieu, R. J. Franklin, M. Schumacher, E. Traiffort, Unexpected central role of the androgen receptor in the spontaneous regeneration of myelin. *Proc. Natl. Acad. Sci. U.S.A.* **113**, 14829–14834 (2016).

37. G. de Monteiro Castro, N. A. Deja, D. Ma, C. Zhao, R. J. Franklin, Astrocyte activation via Stat3 signaling determines the balance of oligodendrocyte versus Schwann cell remyelination *Am. J. Pathol.* **185**, 2431–2440 (2015).

38. C. Devoto, V. A. Guedes, C. Lai, J. J. Leete, S. Mithani, K. Edwards, R. Vorn, B. X. Qu, E. A. Wilde, W. C. Walker, R. Diaz-Arrastia, J. K. Werner, K. Kenney, J. M. Gill, Remote blast-related mild traumatic brain injury is associated with differential expression of exosomal microRNAs identified in neurodegenerative and immunological processes. *Brain Inj.* **36**, 652–661 (2022).

39. M. K. R. Dwyer, N. Amelinez-Robles, I. Polsfuss, K. Herbert, C. Kim, N. Varghese, T. J. Parry, B. Buller, T. A. Verdoorn, C. B. Billing Jr., B. Morrison III, NTS-105 decreased cell death and preserved long-term potentiation in an in vitro model of moderate traumatic brain injury. *Exp. Neurol.* **371**, 114608 (2024).

40. X. Wu, A. J. Levine, p53 and E2F-1 cooperate to mediate apoptosis. *Proc. Natl. Acad. Sci. U.S.A.* **91**, 3602–3606 (1994).

41. G. U. Höglinger, J. J. Breunig, C. Depboylu, C. Rouaux, P. P. Michel, D. Alvarez-Fischer, A. L. Boutillier, J. Degregori, W. H. Oertel, P. Rakic, E. C. Hirsch, S. Hunot, The pRb/E2F cell-cycle pathway mediates cell death in Parkinson's disease. *Proc. Natl. Acad. Sci. U.S.A.* **104**, 3585–3590 (2007).

42. J. Imitola, E. W. Hollingsworth, F. Watanabe, M. Olah, W. Elyaman, S. Starossom, P. Kivisäkk, S. J. Khoury, *Stat1* is an inducible transcriptional repressor of neural stem cells self-renewal program during neuroinflammation. *Front. Cell. Neurosci.* **17**, 1156802 (2023).

43. D. P. Ferrari, M. Bortolanza, E. A. Del Bel, Interferon-γ involvement in the neuroinflammation associated with Parkinson’s disease and L-DOPA-induced dyskinesia. *Neurotox. Res.* **39**, 705–719 (2021).

44. A. C. Kalil, T. F. Patterson, A. K. Mehta, K. M. Tomashek, C. R. Wolfe, V. Ghazaryan, V. C. Marconi, G. M. Ruiz-Palacios, L. Hsieh, S. Kline, V. Tapson, N. M. Iovine, M. K. Jain, D. A.

Sweeney, H. M. El Sahly, A. R. Branche, J. R. Pineda, D. C. Lye, U. Sandkovsky, A. F. Luetkemeyer, S. H. Cohen, R. W. Finberg, P. E. H. Jackson, B. Taiwo, C. I. Paules, H. Arguinchona, N. Erdmann, N. Ahuja, M. Frank, M.-D. Oh, E.-S. Kim, S. Y. Tan, R. A. Mularski, H. Nielsen, P. O. Ponce, B. S. Taylor, L. A. Larson, N. G. Rouphael, Y. Saklawi, V. D. Cantos, E. R. Ko, J. J. Engemann, A. N. Amin, M. Watanabe, J. Billings, M.-C. Elie, R. T. Davey, T. H. Burgess, J. Ferreira, M. Green, M. Makowski, A. Cardoso, S. de Bono, T. Bonnett, M. Proschan, G. A. Deye, W. Dempsey, S. U. Nayak, L. E. Dodd, J. H. Beigel, ACTT-2 Study Group Members, Baricitinib plus remdesivir for hospitalized adults with Covid-19. *N. Engl. J. Med.* **384**, 795–807 (2021).

45. S. Marais, R. P. J. Lai, K. A. Wilkinson, G. Meintjes, A. O'Garra, R. J. Wilkinson, Inflammasome activation underlying central nervous system deterioration in HIV-associated tuberculosis. *J. Infect. Dis.* **215**, 677–686 (2017).

46. H. Wang, Efficacies of treatments for anti-NMDA receptor encephalitis. *Front. Biosci.* **21**, 651–663 (2016).

47. F. Brilot, R. C. Dale, R. C. Selter, V. Grummel, S. R. Kalluri, M. Aslam, V. Busch, D. Zhou, S. Cepok, B. Hemmer, Antibodies to native myelin oligodendrocyte glycoprotein in children with inflammatory demyelinating central nervous system disease. *Ann. Neurol.* **66**, 833–842 (2009).

48. J. Mascarenhas, R. Hoffman, Ruxolitinib: The first FDA approved therapy for the treatment of myelofibrosis. *Clin. Cancer Res.* **18**, 3008–3014 (2012).

49. D. J. Martini, Y. B. Chen, Z. DeFilipp, Recent FDA approvals in the treatment of graft-versus-host disease. *Oncologist* **27,** 685–693 (2022).

50. S. Owji, S. A. Caldas, B. Ungar, Management of atopic dermatitis: Clinical utility of ruxolitinib. *J. Asthma Allergy* **15**, 1527–1537 (2022).

51. A. Sheikh, W. Rafique, R. Owais, F. Malik, E. Ali, FDA approves ruxolitinib (Opzelura) for vitiligo therapy: A breakthrough in the field of dermatology. *Ann. Med. Surg. (Lond)* **81**, 104499 (2022).

52. L. A. Raedler, Jakafi (ruxolitinib): First FDA-approved medication for the treatment of patients with polycythemia vera. *Am. Health Drug Benefits* **8**, 75–79 (2015).

53. Y. Song, F. Zhou, F. Du, Z. Wang, L. Bai, Y. Yao, L. Liu, X. Ma, S. Chen, D. Wu, X. He, Combined emapalumab and ruxolitinib in patients with haemophagocytic Lymphohistiocytosis. *Blood Cancer J.* **14,** 70 (2024).

54. C. Keenan, K. E. Nichols, S. Albeituni, Use of the JAK inhibitor ruxolitinib in the treatment of hemophagocytic lymphohistiocytosis. *Front. Immunol.* **12,** 614704 (2021).

55. L. Scholz, F. Posch, E. Schulz, M. Gornicec, A. Wolfler, A. C. Reisinger, A. Reinisch, P. Eller, F. Eisner, P. Kreuzer, M. Stradner, A. R. Rosenkranz, F. Krammer, G. Schilcher, R. Krause, S. Hatzl, Ruxolitinib, IV immunoglobulin, and high-dose glucocorticoids for critically ill adults with secondary hemophagocytic lymphohistiocytosis: A single-center observational pilot study. *Crit. Care Explor.* **6**, e1046 (2024).

56. H. Qin, J. A. Buckley, X. Li, Y. Liu, T. H. Fox III, G. P. Meares, H. Yu, Z. Yan, A. S. Harms, Y. Li, D. G. Standaert, E. N. Benveniste, Inhibition of the JAK/STAT pathway protects against α-synuclein-induced neuroinflammation and dopaminergic neurodegeneration. *J. Neurosci.* **36**, 5144–5159 (2016).

57. L. Ben Haim, K. Ceyzeriat, M. A. Carrillo-de Sauvage, F. Aubry, G. Auregan, M. Guillermier, M. Ruiz, F. Petit, D. Houitte, E. Faivre, M. Vandesquille, R. Aron-Badin, M. Dhenain, N. Deglon, P. Hantraye, E. Brouillet, G. Bonvento, C. Escartin, The JAK/STAT3 pathway is a common inducer of astrocyte reactivity in Alzheimer's and Huntington's diseases. *J. Neurosci.* **35**, 2817–2829 (2015).

58. L. Ben Haim, M. A. de Carrillo- Sauvage, K. Ceyzeriat, C. Escartin, Elusive roles for reactive astrocytes in neurodegenerative diseases. *Front. Cell. Neurosci.* **9**, 278 (2015).

59. A. Chinnici, L. Beneforti, F. Pegoraro, I. Trambusti, A. Tondo, C. Favre, M. L. Coniglio, E. Sieni, Approaching hemophagocytic lymphohistiocytosis. *Front. Immunol.* **14**, 1210041 (2023).

60. M. Komori, A. Blake, M. Greenwood, Y. C. Lin, P. Kosa, D. Ghazali, P. Winokur, M. Natrajan, S. C. Wuest, E. Romm, A. A. Panackal, P. R. Williamson, T. Wu, B. Bielekova, Cerebrospinal fluid markers reveal intrathecal inflammation in progressive multiple sclerosis. *Ann. Neurol.* **78**, 3–20 (2015).

61. S. H. Anjum, J. E. Bennett, O. Dean, K. A. Marr, D. A. Hammoud, P. R. Williamson, Neuroimaging of Cryptococcal Meningitis in Patients without Human Immunodeficiency Virus: Data from a Multi-Center Cohort Study. *J. Fungi* **9**, 594 (2023).

62. P. Albuquerque, A. M. Nicola, E. Nieves, H. C. Paes, P. R. Williamson, I. Silva-Pereira, A. Casadevall, Quorum sensing-mediated, cell density-dependent regulation of growth and virulence in *Cryptococcus neoformans*. *mBio* **5**, e00986-13 (2013).

63. B. Hommel, A. Sturny-Leclère, S. Volant, N. Veluppillai, M. Duchateau, C. H. Yu, V. Hourdel, H. Varet, M. Matondo, J. R. Perfect, A. Casadevall, F. Dromer, A. Alanio, Cryptococcus neoformans resists to drastic conditions by switching to viable but non-culturable cell phenotype. *PLOS Pathog.* **15**, e1007945 (2019).

64. J. B. Schröder, M. Pawlowski, G. Meyer Zu Hörste C. C. Gross, H. Wiendl, S. G. Meuth, T. Ruck, T. Warnecke, Immune Cell Activation in the Cerebrospinal Fluid of Patients With Parkinson's Disease. *Front. Neurol.* **9**, 1081 (2018).

65. W. B. Haile, C. Gavegnano, S. Tao, Y. Jiang, R. F. Schinazi, W. R. Tyor, The Janus kinase inhibitor ruxolitinib reduces HIV replication in human macrophages and ameliorates HIV encephalitis in a murine model. *Neurobiol. Dis.* **92**, 137–143 (2016).

66. P. Mestdagh, P. Van Vlierberghe, A. De Weer, D. Muth, F. Westermann, F. Speleman, J. Vandesompele, A novel and universal method for microRNA RT-qPCR data normalization. *Genome Biol.* **10**, R64 (2009).

67. E. Y. Chen, C. M. Tan, Y. Kou, Q. Duan, Z. Wang, G. V. Meirelles, N. R. Clark, A. Ma'ayan, Enrichr: Interactive and collaborative HTML5 gene list enrichment analysis tool. *BMC Bioinformatics* **14**, 128 (2013).

68. M. V. Kuleshov, M. R. Jones, A. D. Rouillard, N. F. Fernandez, Q. Duan, Z. Wang, S. Koplev, S. L. Jenkins, K. M. Jagodnik, A. Lachmann, M. G. McDermott, C. D. Monteiro, G. W. Gundersen, A. Ma'ayan, Enrichr: A comprehensive gene set enrichment analysis web server 2016 update. *Nucleic Acids Res.* **44**, W90–W97 (2016).

69. Z. Xie, A. Bailey, M. V. Kuleshov, D. J. B. Clarke, J. E. Evangelista, S. L. Jenkins, A. Lachmann, M. L. Wojciechowicz, E. Kropiwnicki, K. M. Jagodnik, M. Jeon, A. Ma'ayan, Gene Set Knowledge Discovery with Enrichr. *Curr. Protoc.* **1**, e90 (2021).

70. A. D. Rouillard, G. W. Gundersen, N. F. Fernandez, Z. Wang, C. D. Monteiro, M. G. Mc Dermott, A. Ma'ayan, The harmonizome: A collection of processed datasets gathered to serve and mine knowledge about genes and proteins. *Database (Oxford)* **2016**, baw100 (2016).

71. A. Subramanian, P. Tamayo, V. K. Mootha, S. Mukherjee, B. L. Ebert, M. A. Gillette, A. Paulovich, S. L. Pomeroy, T. R. Golub, E. S. Lander, J. P. Mesirov, Gene set enrichment analysis: A knowledge-based approach for interpreting genome-wide expression profiles. *Proc. Natl. Acad. Sci. U.S.A.* **102**, 15545–15550 (2005).

72. Y. Hao, T. Stuart, M. H. Kowalski, S. Choudhary, P. Hoffman, A. Hartman, A. Srivastava, G. Molla, S. Madad, C. Fernandez-Granda, R. Satija, Dictionary learning for integrative, multimodal and scalable single-cell analysis. *Nat. Biotechnol.* **42**, 293–304 (2024).

73. Y. Hao, S. Hao, E. Andersen-Nissen, W. M. Mauck III, S. Zheng, A. Butler, M. J. Lee, A. J. Wilk, C. Darby, M. Zager, P. Hoffman, M. Stoeckius, E. Papalexi, E. P. Mimitou, J. Jain, A. Srivastava, T. Stuart, L. M. Fleming, B. Yeung, A. J. Rogers, J. M. McElrath, C. A. Blish, R. Gottardo, P. Smibert, R. Satija, Integrated analysis of multimodal single-cell data. *Cell* **184**, 3573–3587.e29 (2021).

74. M. P. Emont, C. Jacobs, A. L. Essene, D. Pant, D. Tenen, G. Colleluori, A. Di Vincenzo, A. M. Jorgensen, H. Dashti, A. Stefek, E. McGonagle, S. Strobel, S. Laber, S. Agrawal, G. P. Westcott, A. Kar, M. L. Veregge, A. Gulko, H. Srinivasan, Z. Kramer, E. De Filippis, E. Merkel, J. Ducie, C. G. Boyd, W. Gourash, A. Courcoulas, S. J. Lin, B. T. Lee, D. Morris, A. Tobias, A. V. Khera,

M. Claussnitzer, T. H. Pers, A. Giordano, O. Ashenberg, A. Regev, L. T. Tsai, E. D. Rosen, A single-cell atlas of human and mouse white adipose tissue. *Nature* **603**, 926–933 (2022).

75. D. Schafflick, C. A. Xu, M. Hartlehnert, M. Cole, A. Schulte-Mecklenbeck, T. Lautwein, J. Wolbert, M. Heming, S. G. Meuth, T. Kuhlmann, C. C. Gross, H. Wiendl, N. Yosef, G. Meyer Zu Horste, Integrated single cell analysis of blood and cerebrospinal fluid leukocytes in multiple sclerosis. *Nat. Commun.* **11**, 247 (2020).

76. G. Finak, A. McDavid, M. Yajima, J. Deng, V. Gersuk, A. K. Shalek, C. K. Slichter, H. W. Miller, M. J. McElrath, M. Prlic, P. S. Linsley, R. Gottardo, MAST: A flexible statistical framework for assessing transcriptional changes and characterizing heterogeneity in single-cell RNA sequencing data. *Genome Biol.* **16**, 278 (2015).

77. S. E. Marsh, scCustomize: Custom Visualizations & Functions for Streamlined Analyses of Single Cell Sequencing (2021).

78. S. Højsgaard, U. Halekoh, J. Yan, The R package geepack for generalized estimating equations. *J. Stat. Softw.* **15**, 1–11 (2005).

79. J. C. Hargarten, S. H. Anjum, K. Ssebambulidde, Y. D. Park, M. J. Vaughan, T. L. Scott, D. A. Hammoud, B. J. Billioux, P. R. Williamson, Tocilizumab as a potential adjunctive therapy to corticosteroids in cryptococcal post-infectious inflammatory response syndrome (PIIRS): A report of two cases. *J. Clin. Immunol.* **43**, 2146–2155 (2023).
